# Mental Well-Being or Ill-Being through Coaching in Adult Grassroots Sport: A Systematic Mapping Review

**DOI:** 10.3390/ijerph18126543

**Published:** 2021-06-17

**Authors:** María Rato Barrio, Clemens Ley, Anne Schomöller, Detlef Dumon

**Affiliations:** 1 International Council of Sport Science and Physical Education (ICSSPE/CIEPSS), 14053 Berlin, Germany; mariaratobarrio@yahoo.es (M.R.B.); aschomoeller@icsspe.org (A.S.); ddumon@icsspe.org (D.D.); 2 Institute of Sport Science, University of Vienna, 1150 Vienna, Austria; 3 Department of Health Sciences, FH Campus Wien, University of Applied Sciences, 1100 Vienna, Austria

**Keywords:** mental health, coaching behavior, coach–athlete relationship, trainer, self-determination, health promotion, autonomy support, needs, grassroots, participation coach

## Abstract

There is convincing evidence on the effects of sport and exercise on mental health and well-being. Less evidence is provided about how to achieve these benefits in the context of grassroots sport coaching. We systematically reviewed the scientific literature of three databases to narratively synthesize the current knowledge about which coaching-related factors influence well-being or ill-being, and how to promote mental health in adult athletes through sport coaches. The review includes 52 studies with different methodological and theoretical approaches and mental health outcomes. The wide range of themes were mapped and synthesized within two clusters, i.e., coaching behavior, antecedents, and context; and coach–athlete relationship and social support. The results highlight the importance of the promotion of empowering environments, autonomy-supportive coaching behavior, and coach–athlete relationship quality that relate to the satisfaction of basic psychological needs. The review also calls for a critical perspective, in the sense that the coaching context and working environment may not be empowering and supportive to the well-being of coaches and consequently to the athletes, and that coaches who want to provide autonomy-supportive environments may face various obstacles. Finally, the review synthesizes recommendations for the training of coaches, as one piece of a holistic mental health promotion.

## 1. Introduction

There is convincing evidence on the effects of sport and exercise on mental health and prevention of mental illness. Rebar et al. [1] conducted a meta-meta-analysis based on 92 quantitative studies of the effect of physical activity on depression and anxiety in non-clinical populations, providing the evidence for a medium effect on depression and a small effect on anxiety. Furthermore, Teychenne [2] reviewed the relation between physical activity (PA) and depression and concluded that “even low doses of PA may be protective against depression”. While we also have to account for a placebo effect [mean effect size of 0.20 [95% CI −0.02, 0.41]; [3], as in other interventions, a clear effect [0.37 [95% CI 0.11, 0.63]; [3] of exercise training on psychological outcomes is still evidenced. In addition, the qualitative literature synthesis of Mason and Holt [4] shows the mental health relevant effects and processes of physical activity interventions, particularly regarding social interaction and social support; feeling safe; improved symptoms; a sense of meaning, purpose and achievement; identity and the role of the facilitating personnel. Furthermore, the effects of sport and exercise on various factors of mental well-being, for example, self-esteem [5], self-efficacy [6,7], and resilience [8] were reviewed. Thus, the existing literature provides evidence for the social and mental health benefits of sport and physical activity.

Furthermore, various reviews have synthesized literature on the social and mental benefits of sport participation for children, adolescents and youth [9,10,11,12,13,14,15,16,17,18], adults [1,4,19], and older adults [20,21,22,23]. Together they highlight the importance of considering mental ill-being and well-being, respectively, as barriers and facilitators for sport participation and as outcomes of sport participation. On the one hand, mental health problems and social exclusion negatively influence participation and therefore needs to be thoroughly considered in the promotion of sport participation. On the other hand, these reviews portray potential benefits of sport and physical activity on well-being and social capital. In other words, if one feels well, they are more likely to participate (regularly) in sport and as such benefits (more) from sport participation, improving well-being, which results in a positive, upward spiral effect. If one feels excluded and/or isolates oneself due to ill-being, they are less likely to participate in sport and thus does not benefit from the positive effects of sport on social and mental well-being, favoring a negative, downward spiral effect. Thus, it seems not enough to promote well-being through the positive, upward spiral effect. We should also avoid or interrupt downward spiral effects and care about ill-being in sport.

Less evidence is provided about how to achieve these benefits in the context of recreational grassroots and community sport coaching. For example, Lentz et al. [24] alerted in their review that a “deficient coach-student athlete relationship may lead to mental health symptoms and build over time to an illness in these individuals even after their high school or college athletic career is over”. They furthermore located the need to promote mental health awareness and knowledge in the coaches, considering how the coach establishes and builds relationships with their athletes.

### 1.1. Coaching Context

Importantly, the specific coaching context determines the required knowledge of coaches and athletes’ outcomes, as the coach is required to meet athletes’ needs and help to meet their specific goals [25]. Grassroots sport is defined as “physical leisure activity, organized and non-organized, practised regularly at non-professional level for health, educational or social purposes” [26] (p.98). In contrast to development sport and elite sport, the specific coaching context of recreational grassroots and community sport has a primary focus on participation coaching and less focus on performance coaching.

“*Participation coaching* is distinctive because competition performance is not emphasized, and participants are less intensively engaged with the sport. Objectives are characterized by short-term goals, enjoyment, and health-related outcomes. Performance coaching, on the other hand, entails a more intensive commitment to a preparation program for competition and a planned attempt to influence performance variables. To this end, there is a high degree of specificity in the program that a coach delivers to his or her athletes (e.g., physical conditioning, psychological training)” [25].

Côté and Gilbert further propose objectives for the participation coaching of adults [25]:

- “Provide opportunities for athletes to interact socially
- Afford opportunities for athletes to have fun and playfully compete
- Promote the development of fitness and health-related physical activities
- Teach and assess sport-specific skills in a safe environment for long-term sport involvement
- Teach personal and social assets through sport (citizenship)”

However, grassroots and community sport also includes, to a varying degree, competition and performance orientation. Thus, some of the coaching objectives of a performance coach for adults [25] may be relevant; for example, to prepare athletes for competitive performance and to teach physical, technical, perceptual, and mental skills. 

### 1.2. Purpose and Goals of the Review

While some of the above stated reviews highlight the crucial role of the coach, trainer, or facilitator, these reviews do not specifically focus on the coaching context, behavior, and outcomes on mental well-being. Therefore, we review the scientific literature on coaching and mental well-being to portray the current knowledge about which coaching-related factors influence well-being and how to promote well-being of athletes through coaching. More specifically, we look at how coaching behavior and coach–athlete relationships affect sport participants’ well-being. Thereby, we focus on the *participation coaching* of adult sport participants in grassroots, community, and club sports, excluding high performance orientation and coaching of elite athletes. By doing so, we also aim to contribute to the development of a capacity building approach for coaches promoting well-being-enhancing physical activity and sport offers for adults in the framework of the SPIRIT project. Hence, this review is narrowed to the individual and intrapersonal aspects of a holistic mental health promotion [27]; nevertheless, it aims to understand coaching behavior and coach–athlete relationships within its context of occurrence, antecedents, and outcomes on mental health.

Mental health and wellbeing are both conceptualized from a holistic, salutogenetic perspective, including emotional/affective (e.g., affect balance, happiness, life satisfaction), cognitive (e.g., coping, optimism, hopefulness, sense of personal control), and psychosocial aspects (e.g., social involvement, relatedness, and sociability) and relate to personal growth and psychosocial development [28,29,30]. The holistic approach of both concepts makes a clear delimitation of mental health and mental well-being difficult. For the purpose of this systematic review, we use these two terms interchangeably, aiming to include those literature that use the term mental health as well as those using the term mental well-being.

In order to investigate the role of coaches in the promotion of mental well-being-enhancing physical activity, this review pursues the following objectives:

1. To examine which aspects of mental well-being are addressed in the studies on grassroots sport and physical activity coaching for adults.
2. To explore which types and aspects of the delivery of grassroots-organized sport and physical activity coaching are investigated with regards to the promotion of mental well-being in adults.
3. To map the effects on mental well-being and effect mechanism of the different types and aspects of the delivery of sport and physical activity coaching for adults.
4. To provide recommendations for the training of coaches.

## 2. Materials and Methods

A systematic mapping review was conducted. This approach is convenient to systematically review the literature on coaching and mental well-being regarding the before-stated four objectives, and to categorize and map the wide range of themes and studies accordingly [31].

### 2.1. Search Strategy

Firstly, a preliminary search was conducted to identify possible systematic reviews previously carried out on the subject. Then, final keyword combinations were applied in three databases, i.e., Sportdiscus, PsychInfo, and PsychArticles, until July 2020. The search strategy combined search terms relating to sports, physical activity, or exercise AND terms relating to mental/emotional/psychosocial wellbeing or health AND terms relating to coach/trainer, but NOT rehabilitation, therapy, patient, elite athlete, or high performance. Studies published from January 2005 to July 2020 in English, Spanish, Catalan, French, German, Italian, and Portuguese in peer reviewed journals were included. With regards to age, the general adult population (>18 years) was considered. As described above we focused on participation coaching and excluded articles on performance coaching whenever they dealt with high performance and elite athletes/coaches. When a study included various levels of participation or performance, the study was excluded when the sample group consisted of more than one third of high-level athletes. The inclusion and exclusion criteria are outlined in Table 1.

### 2.2. Study Selection Process

**Figure 1:**
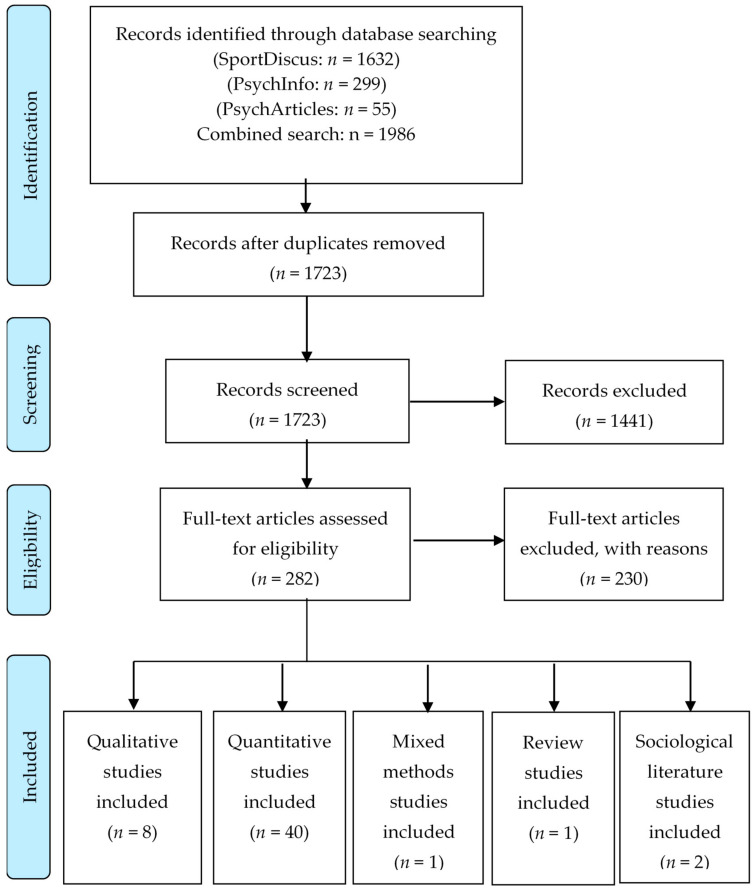
PRISMA flow chart.

MRB conducted the search in the four different databases and removed duplicates. Following this, MRB and CL both independently screened the titles and abstracts of the resulting studies (see Figure 1 Flow chart). After removing the excluded studies, they also independently screened the full texts of the remaining studies in an unblinded standardized manner. Reasons for exclusion were noted in the spreadsheet. Any discrepancy was discussed and resolved by consensus. 

### 2.3. Data Extraction and Analysis

Based on the PRISMA recommendations [32], a spreadsheet was created with the following criteria for the data extraction: reference; cluster and theme; objective(s) of the study and study design; sample, including size, type of participants, gender, age, sport(s) performed by the participants, level of the performance; country of study; intervention, setting and time frame; outcomes, categories analyzed and measurement instruments; key findings and further comments. MRB and CL independently assessed all papers and extracted the data. Similar to O’Driscoll et al. [33], no formal assessment of methodological quality of individual studies was undertaken with a checklist in this review. However, while assessing all papers, we appraised the study quality and the risk of bias of the studies in the research clusters, which we used in the synthesis of the results (see Section 3.5). In accordance with the study objectives (see Section 1.2) this decision was made due to the heterogeneity of the methodological approaches used in the included studies. These 52 studies included diverse cross-sectional and longitudinal study designs and applied qualitative, quantitative, or mixed methods as well (see Section 3.3.). We are aware of the limitations of this approach (see Section 4.4); however, we prioritized the broader scope in this mapping review, including different methodological approaches.

**Figure 2:**
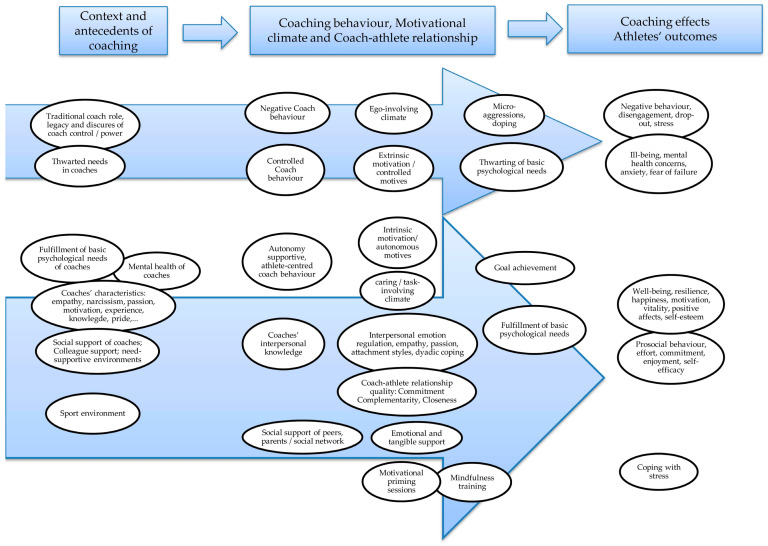
Conceptual map of themes according to coaching context, behavior and outcome on mental well-being.

During data extraction, main themes were identified and then mapped (see Figure 2).

Further analysis was guided by the following questions:

- Which aspects of mental well-being are addressed in the studies on grassroots-organized sport and physical activity coaching for adults?
- Which types and aspects of the delivery of grassroots-organized sport and physical activity coaching are investigated with regards to the promotion of adults’ mental well-being?
- What are the effects on mental well-being and effect mechanism of the different types and aspects of the delivery of sport and physical activity coaching for adults?
- Which recommendations can be drafted for mental health promotion through coaching and for the training of coaches?

In the following sections, the results of the study selection and the description of the study characteristics are presented, succeeded by a conceptual map of themes and the synthesis of results. 

## 3. Results

### 3.1. Study Selection Results

From the 1723 records screened, 282 were eligible for full-text assessment. These 282 full-text articles were assessed using the inclusion and exclusion criteria (see Table 1). A total of 230 studies were excluded. The reasons for exclusion were: outcomes or type of sport/intervention out of scope (*n* = 184); age of participants (*n* = 24); level of performance (*n* = 5); type of publication/study design (*n* = 9); and no full-text article available (*n* = 9). Thus, the review included 52 studies, which were classified into two thematic clusters:

- Coaching behavior, antecedents, and context (*n* = 32)
- Coach–athlete relationship and social support (*n* = 20)

### 3.2. Study Characteristics

The selected studies included coaches (*n* = 6327) and athletes (*n* = 9677). Studies were normally gender-mixed, with seven exceptions of which five were studies with a 100% female sample and two had a 100% male sample. The age range was 18–80, including the total period of adulthood and older adulthood. The samples included sport coaches and participants from a wide range of individual and team sports and exercise programs (see also Supplementary Materials).

The studies were from a broad sample of countries. The majority of the studies came from the United Kingdom (*n* = 22), followed by the United States of America (*n* = 10), and then by Canada (*n* = 4) and Spain (*n* = 4). We also found a couple of studies from Norway (*n* = 2) and Belgium (*n* = 2). France (*n* = 1), Hungary (*n* = 1), Lithuania (*n* = 1), Sweden (*n* = 1), Switzerland (*n* = 1), and the Netherlands (*n* = 1) in Europe; Japan (*n* = 1), Singapore (*n* = 1) and China (*n* = 1) in Asia; and New Zealand (*n* = 1) in Oceania, were also represented.

### 3.3. Method Characteristics

The review included a total of 40 quantitative research studies, 8 qualitative studies, 1 used mixed methods, 1 reviews, and 2 sociological literature studies. The predominant quantitative approach was cross-sectional (*n* = 33), particularly to test predictors of athletes’ outcomes and process models, analyzing the paths from coach characteristics, coaching behavior, and coach–athlete relationship to athletes’ well-being. Furthermore, four quantitative, longitudinal studies were included, involving one randomized controlled trial. Validated questionnaires were the most common research instrument of the quantitative studies (see also Supplementary Materials).

The qualitative studies used different ontologies and approaches, including constructivist and post-positivist assumptions, grounded theory, and phenomenological approaches. The qualitative studies mostly used interviews, focus groups, field diaries, and observation. Qualitative data analysis often included thematic analysis.

### 3.4. Outcome Measures: Mental Well-Being

The different studies measured the outcome of mental well-being in quite different ways. The main indicators for mental well-being often included happiness, (life) satisfaction, and resilience as well as self-esteem, self-concept, self-confidence, self-efficacy, and body image/satisfaction. Stebbings et al. [34] differentiated between hedonic well-being, which particularly refers to positive affect, happiness or pleasure, and eudaimonic well-being, which is defined as self-actualization, personal growth, and congruency between personal and occupational roles, values, beliefs, and identity [35]. A few studies used other concepts, such as flow experience [36,37] or sense of coherence [38], to indicate mental well-being. Some studies measured the negative outcomes on well-being (ill-being) through the prevalence of disorders, burnout, depression, anxiety and fear of failure, and negative affect [39,40,41,42,43,44,45]. Blood cortisol levels were also used as a marker of psychophysiological stress [46]. Aspects relating to the social well-being were prosocial and antisocial behavior and social cohesion [47,48,49]. Furthermore, the basic psychological needs (i.e., sense of autonomy, competence, and relatedness) are related to well-being through the self-determination theory [39,40,41,42,43,44,47,48,50,51,52,53,54,55,56,57]. Effort, commitment, and enjoyment were used to measure positive coach–athlete relationship outcomes, and closeness, commitment, and complementarity as indicators of coach–athlete relationship quality [57,58,59,60,61].

### 3.5. Mapping of Results

Figure 2 illustrates the main themes identified in the included studies. The themes were mapped according to the antecedents and context of coaching, behavior, and outcome on mental well-being, in order to illustrate possible processes and paths of interaction. However, the conceptual map is simplified and does not represent all possible interactions. These paths are not necessarily linear and unidirectional, as the interactions of the themes can be multidimensional and reciprocal. Furthermore, most studies were cross-sectional and do not provide proof for the directional effects. Thus, the arrows in Figure 2 rather mirror the theoretical concepts and considerations found in the included studies.

### 3.6. Synthesis of Evidence

The results of these themes were synthesized (see also Supplementary Materials) according to the two overarching clusters:

1. Coaching behavior, antecedents, and context
2. Coach–athlete relationship and social support

#### 3.6.1. Studies on Coaching Behavior, Antecedents, and Context

Many studies of this cluster analyzed the effects of autonomy-supportive versus controlled coaching behavior [34,36,39,40,41,43,44,45,47,48,50,51,52,53,54,62,63,64,65]. In these studies, the theoretical foundations were the self-concordance model and the self-determination theory with its subtheories on motivational regulation and basic psychological needs (i.e., autonomy, competence, and relatedness). The studies in this cluster are mainly cross-sectional, analyzing associations of the coaching behavior with satisfaction or thwarting of basic psychological needs, self-determined motivation, autonomous motives, and finally well-being outcomes, testing the self-determination theory [39,40,41,43,44,47,48,50,51,52,53,54]. These studies consistently proved the self-determination theory as a strong and meaningful theoretical foundation. These cross-sectional studies were mainly of a high quality and a low risk of bias [39,40,41,43,44,47,48,50,52,53,54]. However, they do not allow for cause-effect relationship analysis. The testing of relations, effects, or predictors is theory-driven. There is a scarcity of longitudinal studies, using both quantitative (preferably, randomized control trials) and qualitative (e.g., qualitative experiments or multiple case studies) research designs, to analyze cause-effect relationships.

Furthermore, less is known on the antecedents for autonomy-supportive coach behavior and the context in which it can take place [66]. The latter is an important issue as the coaching context is not necessarily and traditionally autonomy-supportive [67]. From a critical perspective, various studies showcased poor coaching and negative effects of coaching on well-being [68,69,70,71,72,73]. These studies included three qualitative studies of a high quality [68,69,72] and one qualitative study showing lower quality in the methodological procedures and reporting [70]; and one cross-sectional [71] and one longitudinal study with a higher risk of bias due to the nature of the study [73]. The few studies on coach characteristics and antecedents of autonomy-supportive (versus controlled) coaching behavior were conducted in relation to coaches’ narcissism and empathy; harmonious (versus obsessive) passion; coaches’ well-being, need satisfaction and motivation; as well as affective states, self-awareness, and emotional regulation [34,45,60,63,64,65,74]. While limited in regards to their cross-sectional design, these studies showed a high quality in the methodological procedures and reporting [45,60,63,64,65,74]. Furthermore, Stebbings et al. used a longitudinal design with three measurement points to analyze the effects of well- and ill-being on coaches’ autonomy supportive and controlling behaviors over time [34].

#### 3.6.2. Studies on the Coach–Athlete Relationship and Social Support

Three studies focused on the coach–athlete relationship quality (i.e., closeness, commitment, and complementarity) in regards to indicators of well- or ill-being such as athletes’ stress appraisal and management, fear of failure, needs satisfaction, vitality, and cohesion [55,58,61]. Another topic was the style of coach–athlete attachment. The attachment style was investigated in relation to positive and negative affect and need satisfaction [55,56,58]. These cross-sectional studies showed a high quality in the methodological procedures and reporting. A qualitative longitudinal multiple case study on the coach–athlete relationship addressed interpersonal emotion regulation [75]. In addition, several studies analyzed the support from coaches, peer mentors, and teammates for athletes’ mental health [48,49,76,77,78]. The latter involves informational, emotional, and esteem support as well as social network and tangible support [79].

## 4. Discussion

There is convincing evidence on the effects of sport and exercise on mental health and well-being [1,2,4,5,6,9,19]. Less evidence is provided about how to achieve these benefits in the context of grassroots sport coaching. We systematically reviewed the scientific literature of three databases to narratively synthesize the current knowledge about which coaching-related factors influence (positively and negatively) well-being and how to promote mental health in grassroots sport through coaching. In the following, we discuss the results according to the four proposed study objectives.

The included 52 studies addressed diverse aspects of well-being (objective 1), including positive affect, different facets of self-concept, cohesion, and the satisfaction of basic psychological needs. Ill-being was measured through the prevalence of mental disorders but also through negative affect. This wide operationalization of mental being resonate with the existing definitions of well-being and mental health [29,30], which encompass more than the absence of mental disorders or illness. The studies showcase a rather salutogenetic stance, focusing on the resources and the factors that maintain a healthy person, instead of a pathogenetic approach, which searches for the causes of illness [28]. Such a holistic and salutogenetic approach is suitable for the promotion of mental health in the coaching context of grassroots sport and is in line with the above presented objectives for participation coaching, proposed by Côté and Gilberts [25].

Furthermore, the review synthesizes various types and aspects of the delivery of grassroots sport coaching (objective 2) and their effects on mental wellbeing (objective 3). These aspects of delivery and potential effect mechanisms are discussed in the following according to the two overarching clusters, i.e., coaching behavior, antecedents, and context, and coach–athlete relationship and social support. Finally, recommendations for the training of coaches, as an integral part of the review (objective 4) are discussed.

### 4.1. Coaching Behavior, Antecedents, and Context

The reviewed studies analyzed, above all, the effects of autonomy-supportive versus controlled coaching behavior on satisfaction versus thwarting of basic psychological needs of athletes (i.e., autonomy, competence, and relatedness) [39,40,41,43,44,47,48,50,51,52,53,54]. Controlled coaching behavior is associated with needs thwarting, which leads to ill-being or negative health outcomes such as eating disorders, burnout, depression, negative affect, physical symptoms, perturbed physiological arousal, and antisocial behavior [39,40,41,47,53,62]. In contrast, autonomy-supportive coaching behavior is associated with need satisfaction; need satisfaction in turn is associated with various mental well-being outcomes, e.g., positive affect, vitality and motivation, life satisfaction, resilience, and self-concept as well as prosocial behavior towards teammates. Several studies showed that athletes’ perception of autonomy-supportive coaches predicts athletes’ perceived competence, autonomy, and sense of relatedness, which, in turn, predicts motivational regulation (i.e., autonomous motivation) [48,50,54], and finally, greater self-esteem and life satisfaction [48,51,53,54], vitality and positive affect [40], greater sport satisfaction and positive emotions in sports [52], as well as athletes’ prosocial behavior towards teammates [47]. These interactions seem invariant across gender and level of competition [50] and are evidenced in non-competitive exercise and fitness programs as well [48,53,54].

Besides autonomous motivation [48,50,54], athletes’ implicit disposition towards autonomy [36], goal motives, efforts, and goal attainment [43,44,62], and the motivational climate [46,80] seem to moderate or mediate the relationship between autonomy-supportive coaching behavior and well-being. For example, autonomy-supportive coaching behavior seems to predict autonomous goal motives, which, in turn, relate to effort; effort to goal attainment; goal attainment to need satisfaction; and need satisfaction to psychological well-being [43,44,62]. Schüler et al. [36] showed that individuals’ implicit disposition towards autonomy influence the degree to which people benefit from autonomy need satisfaction. Finally, athletes’ perceptions of autonomy-supportive coaching behavior positively predicted a task-involving and empowering climate, which relates to adaptive behavior correlations. Athletes’ perceptions of controlling behavior predicted an ego-involving and disempowering climate, and thus maladaptive behavior correlates [46,80,81].

Several antecedents and coach characteristics were studied in relation to autonomy-supportive versus controlled coaching behavior. Autonomy-supportive coaching behavior was related to coaches’ empathy, harmonious passion, as well as positive affective states, self-awareness and emotional regulation. Matosic et al. [63] showed a positive direct relation between coaches’ narcissism and controlling coach behavior as well as an indirect relation mediated by empathy. Lafrenière et al. [74] showed that harmonious passion predicted coaches’ autonomy-supportive behaviors, whereas obsessive passion predicted controlling behavior.

Furthermore, the need satisfaction and well-being of the coach impacted upon coaching behavior and interpersonal behavior towards athletes [34,45,65]. Opportunities for professional development, job security, and work-life balance predicted coaches’ need satisfaction, positive affects, and subjective vitality [45]. In turn, coaches’ well-being and positive affects is associated with autonomy support toward the athletes [34,65]. However, coaches’ well-being is endangered by occupational stressful experiences, traumatic events, and a variety of performance-related, organizational, contextual, interpersonal, and intrapersonal stressors [42]. The working environment and the coaching context is not necessarily autonomy-supportive; sport coaching is traditionally rather controlled, taking place in a disciplinary environment [67], which is rather thwarting of basic psychological needs and well-being. Based on Foucault, Denison et al. [67] asks how to achieve the desired outcomes of autonomy-supportive and empowering coaching behavior in a traditional controlled and disciplinary framework that normalizes maximum coach control in sports. The authors argue that this change in coaching behavior needs to be accompanied by changes to the power relations and by a critique of sports’ disciplinary legacy, which includes “techniques and instruments of discipline associated with the military, work and particularly the prison” (p. 780). “Given that power can be both restrictive and productive, and that discipline can be both limiting and enabling, it can be extremely challenging for a coach to begin coaching in a way that affords opportunity and choice when needed and constraint and control when needed” (p. 780).

Negative coaching behaviors are also reported. For example, athletes described how coaches were inhibiting athlete’s mental skills by distracting, engendering self-doubt, demotivating, and dividing the team [68]. Gearity and Metzger [69] describe micro-aggressions in men’s sport coaching at the intersection of sport coaching, mental health, and social identities. Aicinena [70] argues that coaches, athletes, and parents exhibit hubristic pride that causes harm to others. Hillier et al. [71] describe the coaches as the primary source of influence with regards to the rapid weight loss in professional and amateur mixed martial arts athletes, which has negative implications on athletes’ well-being.

Furthermore, characteristics of the sport or the sport practice in itself may promote positive (e.g., value-based) or negative (e.g., aggressive) environments that favor respective behaviors (e.g., micro-aggressions, hubristic pride) [72,73]. Thus, the coach must be aware of potential negative behaviors and values inherent to the respective sport practice and build safe coaching environments.

Inherent to many sport practices is the increased attention paid to the body and physical appearance. While body satisfaction is important for health [38], coaches should “encourage a culture that focuses less on body appearance and more on cultivating positive body image” [37]. An improved body image can positively influence and stabilize self-esteem [82], and improve self-worth, promoting adherence to physical activity [83].

While this review voiced some critical opinions and potential negative aspects of sport practices and coaching, various specific systematic reviews have focused on other negative aspects in sport, e.g., exercise addiction and eating disorders [84,85,86], aggression and violence in sport [87]. Recently, concussion in sport and the association with mental health and depression was investigated [88,89].

### 4.2. Coach–Athlete Relationship and Social Support

Supportive coaching behavior is also associated with the quality of the coach–athlete relationship (i.e., closeness, commitment, and complementarity), which in turn relates to athletes’ well-being [60,61,74,90], stress appraisal, and coping mechanisms [90] as well as to reduced fear of failure [61]. Closeness in the coach–athlete relationship was associated with challenge appraisals positively and with threat appraisals negatively. Rather surprisingly, commitment in CAR was positively associated with threat [90]. The authors indicated that “although it is important that both the coach and the athlete are committed to the relationship, coaches could speak to their athletes and provide re-assurances about factors that might cause threat (e.g., the outcome of competitions) in highly committed coach–athlete relationships” (p. 25). In addition, athletes are less likely to experience fear or shame and embarrassment upon failure if they perceive their coach to be empathic towards them [61].

The coach–athlete relationship quality is related to athletes’ attachment styles and perceived affect [58]. The secure attachment style is associated positively with social support and relationship depth, and negatively with interpersonal conflict, which consequently influence athletes’ affect negatively [58]. Multiple studies of Felton and Jowett [55,56,57,59] showed that the relationship between attachment style and well-being was mediated by athletes’ need satisfaction. This is an important point as “even athletes with an avoidant attachment style are more likely to feel that their potential is realised if their needs are satisfied within the coaching relational context” [55] (p. 62). In another study, Felton and Jowett [56] showed that thwarted autonomy and competence needs mediated the relation between athletes’ perceived insecure attachments to the coach and life satisfaction and negative affect. Furthermore, thwarted competence and relatedness needs mediated the relationship between athletes’ perceived attachment style and experiences of performance satisfaction, life satisfaction, depression, and negative affect [57]. Furthermore, need satisfaction mediated the effects of complementarity on vitality and on task and social cohesion [59]. Perceived cohesiveness positively predicted the satisfaction of basic needs, particularly the relatedness need [52].

Furthermore, interpersonal emotion regulation was investigated in the coach–athlete relationship. Coaches’ emotions (e.g., happiness and anger) influence athletes’ emotions [75,91]. Braun et al. [75] provides interesting insights on interpersonal emotion regulation in individual sports through a qualitative, longitudinal multiple case study with five cases; including in each case, one coach and two of his varsity sport athletes. Closeness within a coach–athlete dyad seemed to favor coaches’ attempts to regulate their athletes’ emotions, and vice versa [75].

Lafrenière and colleagues [60,74] analyzed the role of passion for sports for the coach–athlete relationship quality, using the Dualistic Model of Passion. Positive emotions mediated the effects of harmonious passion on the coach–athlete relationship quality, which in turn predicted coaches’ well-being [60]. Furthermore, harmonious passion predicted coaches’ autonomy-supportive behaviors, which in turn predicted high quality coach–athlete relationships, resulting in athletes’ general happiness [74].

While some studies investigated the coach–athlete relationship from a coach’s perspective or from an athlete’s perspective, various studies opted for a dyadic approach, investigating coach–athlete pairs with an interpersonal perspective. Staff et al. [92] describe the essence, antecedents (lock and key fit, friendship and trust, communication of the stressors) and outcomes (protection and support) of dyadic coping. Nicholls and Perry [93] detected that relationship quality was particularly important for coaches, but less important for athletes.

Stefansen et al. [94] conducted twenty gender-mixed focus group interviews with sport students, using four short films as a common starting point for exploring their thinking about coach–athlete sexual relationships (CASRs). On the one side coach–athlete sexual relationships are viewed as ethically problematic and on the other side as acceptable. The findings revealed that “three different ethics were activated in the interviews: the safeguarding, love, and athletic-performance ethics”. Finally, the authors offer thoughts for sporting organisations’ prevention efforts.

Finally, several studies showed the benefits of informational and tangible social support from coaches as well as from peer mentors and teammates on athletes’ well-being, showing the synergies and benefits of support from various, different providers [49,77,78,95,96].

### 4.3. Practical Implications and Recommendations for the Training of Coaches

The selected studies yielded several recommendations. The studies consent the promotion of autonomy-supportive, empowering environments; e.g., “coaches should use behaviours that support their athletes’ autonomy in relation to their personal goals. For example, such behaviours can be demonstrated by providing a sense of choice and adopting their athletes’ perspectives, while also avoiding behaviours, such as the use of controlling language, which may exert external, or encourage internal, pressures” (Smith et al., 2010, p. 31). However, Denison et al. [67] questions how to achieve the desired outcomes of autonomy-supportive and empowering coaching behavior in a traditional controlled and disciplinary framework that normalizes maximum coach control in sports. The authors argue that this change in coaching behavior needs to be accompanied by changes to the power relations and by a critique of the sports’ disciplinary legacy. Thus, *coaching differently* (what the authors call the empowering, autonomy-supportive coaching behavior) means “to continually problematize what they do—the details of the practices they consistently follow, the types of relationships they form—and what they say—the metaphors, analogies and examples they use, the instructions they give, the questions they ask, the points they emphasize and of course the questions and points they do not ask or emphasize” [67]. Thus, a (self-)reflective stance is required. Another critical theme is body image, as the body is particularly present and exposed in sports. Soulliard et al. [37] advise “to encourage a culture that focuses less on body appearance and more on cultivating positive body image” and “to deliver messages of appreciation for their athletes’ bodies with a particular focus on how their bodies allow them to perform successfully in their sport”. Huberty et al. [83] recommend “improving or deemphasizing body image” in order to improve self-worth.

The coaching environment also has implications on the well-being of the coach, and thus on their behavior and consequently on athletes’ well-being. Opportunities for professional development, job security, and work-life balance are important to facilitate coaches’ need satisfaction and well-being [45]. Coaches’ working environments could be examined in terms of being supportive to the eudaimonic well-being of the coach as well as, for example, promoting integration of the coaching role with their own values (integration) and supporting autonomous motivation [34]. “When coaches experience a sense of congruence between their coaching role and their personal values, this may empower them with more energy to invest personal time and effort into that role. This implies that performance directors, head coaches, and other employers of coaches should allow coaches the freedom to express their ideas and work in accordance with their values and beliefs. This can be achieved by providing choice and avoiding strict regulation of management and leadership strategies” [34]. Furthermore, educational programs of coaches should include emotional regulation, self-awareness, and mindfulness training to improve coaches’ regulation of affective states in coaching athletes.

Koh et al. [49] provide important insights for coaches on how to incorporate social support strategies into coaching to better support athletes’ well-being. They distinguish key strategies for emotional support (e.g., showing genuine concern in athletes’ well-being or maintaining continuous support by being available to athletes), esteem support (e.g., teaching athletes techniques to deal with pressure, providing positive reinforcements to athletes or building athletes’ confidence through self-discovery of techniques), informational support (e.g., providing contextual feedback, helping athletes reflect on their performance or understanding athletes’ goals), and tangible support (e.g., providing practical help to reduce athletes’ worries and stress or helping athletes explore new opportunities).

Noteworthy to mention in the context of this discussion are the following three reviews, which border on the scope of our review. Bissett et al. [97] conducted a narrative review of literature and a Delphi-study on the role of coaches in the prevention of athletes’ mental illness, framed within the World Health Organization’s prevention framework. Accordingly, they concluded potential coach behaviors for primary (e.g., coaches should not use language that stigmatizes mental illness and mental health help-seeking; coaches should positively reinforce athlete behaviors that are consistent with a team culture supportive of mental health and mental health help-seeking), secondary (e.g., coaches should attend to changes in athlete behavior that may indicate the emergence of a mental health concern; if coaches think an athlete may be an immediate threat to the safety of others, coaches should contact emergency services), and tertiary prevention (e.g., coaches should protect the confidentiality of athletes’ mental health help-seeking, consistent with athletes’ preferences; coaches should continue to offer athletes opportunities for engagement in team activities if athletes are taking a break from competition due to mental health concerns). Breslin et al. [98] synthesized quantitative studies on interventions that aim to promote mental health awareness and well-being in coaches and athletes. The review included a substantial heterogeneity of studies (with limited validity of measures), participants, and interventions. Nevertheless, they portray some potential effects of interventions on mental health knowledge, stigma, referral efficacy, help-seeking intentions and behavior, and well-being outcomes (self-concept, depressive symptoms, negative affect, mental toughness, relationships, and substance abuse). The authors finally call for evidence and theory-based intervention programs designed to increase mental health literacy and to promote the well-being of athletes, coaches, and officials. They recommend using psychological theories, specifically, the Self-Determination Theory [99] and the Theory of Planned Behavior [100] for designing such interventions. Geidne et al. [27] conducted a systematic mapping review, providing useful guidelines for a setting-based approach in health promotion in sport clubs. This approach takes a more socio-ecological stance, emphasizing that multiple levels of influences exist, interact, and should be addressed in health promotion initiatives. As our review aims to provide recommendations for the training of grassroots sport coaches, we have to acknowledge that the capacity building approach should only be considered as one piece of an integral mental health promotion initiative. 

### 4.4. Limitations

The definitions and terms used are social-culturally influenced. Terms such as sport or mental health have varying meanings in different *cultures* and settings (depending, e.g., on the socio-political climate, historical context) [30]. We acknowledge that the definitions and concepts presented here are mostly influenced by literature from socioeconomically developed countries and rather individualistic societies (e.g., Europe, Northern America, Australia).

The included studies showcase a broad range of methodological approaches and quality as well as diverse outcome measures of mental well-being. As a consequence, no meta-analysis was conducted. The lack of longitudinal studies does not allow building evidence on cause-effect relationships. However, many of the reviewed studies provide a strong theoretical foundation.

The scope and search strategies of this systematic mapping review were quite broad. This approach resulted in a good overview and conceptual map of prevailing topics and studies in the field of coaching related to mental well-being. The included studies provided findings from different perspectives and methodological approaches that were complementary to each other and provided a more comprehensive picture (see Section 3.4.). However, the mapping review approach takes the risk to “simplify the picture and mask considerable variation (heterogeneity) between studies and their findings” [31] (p.98), for example, the diverse quality of and risk of bias in the different studies (see Section 3.5.). Furthermore, the inclusion of only three databases may have resulted in the absence of other relevant studies that are indexed only in other databases. In addition, the exclusive focus on adults resulted in the exclusion of some studies that—although targeting a different age—may have possibly been informative for coaches of adults as well. Similarly, the search strategy excluded studies on high performance and coaching of elite athletes that may have provided some extent aspects that are independent of the coaching context. As discussed in the introduction, participation coaching and recreational grassroots sports do include competition and performance orientation and the degree may differ among sports, clubs, and players levels. Furthermore, performance coaching and participation coaching are also two overlapping concepts (see Section 1). The distinction between grassroots sports and high performance is diffuse, as competition and performance gradually increases. Although professional sports and high performance coaching is not targeted in this review, it is worth mentioning that the International Society of Sport Psychology published a position paper on athletes’ mental health [101]. The authors outline that “mental health is a major resource for athletes in relation to their performance and development”, but “athletes experience additional mental health risk factors compared to non-athletic population, such as high training loads, tough competitions, and a stressful lifestyle” (p. 622) and mental health-related problems, such as concussion, overtraining or identity crisis. The position paper concludes with various postulations and challenges, including the call “to contribute to the development of autonomy supportive and culturally safe athletic environments at all sport levels; to work on increasing cultural competences of athletes and staff to destigmatise minority and migrant athletes, and facilitate sharing between cultures; to work systematically on increasing athletes’ and coaches’ mental health literacy, and destigmatising mental health interventions” (p. 633). Many of these aspects seem important for promoting well-being in grassroots, community, and club sport as well, and resonate in the results of this review.

## 5. Conclusions

The results of the review provide valuable understandings for the promotion of mental well-being in grassroots sport and for the development of a capacity building approach for coaches to promote mental health-enhancing sport for adults. Various aspects of delivery and potential effect mechanism of sport coaching on mental well-being and ill-being were discussed and the theoretical concepts mapped (see Figure 2). The review highlights the importance of considering coaching behavior and the coach–athlete relationship from mental health and well-being perspectives. This includes the facilitation of autonomy-supportive environments, satisfaction of basic psychological needs (i.e., autonomy, competence, and relatedness) and coach–athlete relationship quality and social support. Autonomy-supportive coaching behavior is associated with need satisfaction, which in turn is associated with various mental well-being outcomes, e.g., positive affect, vitality and motivation, life satisfaction, resilience, and self-concept as well as prosocial behavior towards teammates. Autonomy-supportive coaching behavior is also associated with the quality of the coach–athlete relationship, which in turn relates to athletes’ well-being, stress appraisal, and coping mechanisms. In contrast, controlled coaching behavior is associated with needs thwarting, which leads to ill-being or negative health outcomes such as eating disorders, burnout, depression, negative affect, physical symptoms, perturbed physiological arousal, and antisocial behavior. Thus, the coach should promote an empowering climate, but also avoid controlling behavior and needs thwarting. Therefore, a continuous, (self-)reflective stance is required, as described above in the practical implications and recommendations for the training of coaches. Considering these implications, each coach should contribute, on the one hand, to the promotion of the positive, upward spiral effects described at the beginning of this article, fostering sport participation and benefits on well-being. On the other hand, each coach must contribute to the avoidance or interruption of the negative, downward spiral effects, by reducing controlled environments, poor coach–athlete relationship quality, and thwarting of basic psychological needs.

The review also calls for a critical, multi-level perspective, in the sense that the coaching context and working environment may not be empowering and supportive to well-being, and that coaches, who want to provide autonomy-supportive environments, may face various obstacles, e.g., in relation to expectations from athletes and other coaches and customs of traditional coaching practices. Thus, it seems not enough to promote well-being through the positive, upward spiral effect. We should also avoid or interrupt downward spiral effects and care about ill-being in sport because it prevents achievement of the above described health benefits.

Putting the recommendations into practice, mental health promotion in the context of grassroots sport coaching should be implemented at various levels, including:

- Improving coaches’ occupational environment and coaching context as well as their well-being (e.g., building supportive working environments, favoring coaches’ needs satisfaction and self-determined motivation, hedonic and eudaimonic well-being, and avoiding thwarting of coaches’ needs; stimulating critical thinking and awareness of stressors and resources for coaches’ well-being).
- Stimulating coaches in reflecting on and improving of own coaching behavior and coach–athlete relationship quality with regards to effects on mental well-being and ill-being (e.g., how to build autonomy-supportive environments, how to favor athletes’ needs satisfaction and self-determined motivation, and to avoid thwarting of athletes’ needs; to stimulate critical thinking and awareness of stressors and resources for athletes’ well-being).
- Supporting coaches and peer leaders in the promotion of athletes’ well-being and reducing ill-being (e.g., through capacity-building workshops, supportive infrastructure, and social support).

Implementation studies should analyze putting the recommendations into practice. Respective mental health promotion initiatives should be evaluated in the context of grassroots sport coaching. For example, it is worth analyzing if and how capacity-building initiatives influence the actual coaching behavior and the coach–athlete relationship quality, and finally impact on athletes’ well-being [102]. The effective transfer of coaches’ knowledge from mental health capacity-building to the athletes seems a practical challenge and requires a better understanding of transfer processes [103,104]. Furthermore, it is of particular interest to improve understanding of how to achieve autonomy-supportive coaching behavior and empowering climates in a traditional sporting context, which normalizes maximum coach control in sports [67]. This underlines the importance of conducting holistic, multi-level research to analyze the various factors and contexts that influence coaches’ and athletes’ behavior and well-being. The included studies consistently proved the self-determination theory as a strong and meaningful theoretical foundation. Further research should build upon this foundation and investigate the delivery and effects of grassroots sport coaching on mental health through conducting longitudinal studies, using quantitative and qualitative methods, and experimental study designs.

## Figures and Tables

**Table 1:** Inclusion and exclusion criteria.

	Inclusion Criteria	Exclusion Criteria
Study design	Quantitative studies; qualitative studies; literature studies; reviews	Commentaries; viewpoints; case reports; protocols; development and/or validation of questionnaires
Type of publication	Peer-reviewed journal articles	Not peer-reviewed Books, book chapters
Year of publication	2005–2020	Before 2005
Language	English, French, German, Italian, Portuguese, Spanish, and Catalan	
Population	Coaches/trainers and athletes	Elite athletes; patients; people with an illness, disorder or clinical condition; injured athletes
Age	Adults (18 years and older)	Under 18 years
Type of sport (intervention)	Recreational, community, and grassroots sport; organized sport, exercise and physical activity programs.	High performance; sport as therapy, rehabilitation or recovery; non- or self-organized physical activity, recreation or leisure (not sport or exercise specific)
Type of intervention	Coaching; (educational) programs that aim to promote mental well-being of coaches and/or athletes	No reference to coaching or coaches/trainers/facilitators; programs that aim to promote physical activity levels
Outcome (see also search strategies)	Outcomes related to mental/psychological well-being or mental health; psychosocial outcomes (e.g., affect, psychological functioning, psychological needs, self-concept, resilience); coach behavior; coach–athlete relationship quality.	Outcomes related to performance; competition-related anxiety or stress; injury or injury recovery; physical activity motivation and adherence; physical activity level; outcomes related to physical health or physical well-being or physical functioning; therapeutic outcomes; concussion; mental toughness.

## Data Availability

Not applicable.

## Supplementary Materials

The following are available online at https://www.mdpi.com/article/10.3390/ijerph18126543/s1, Table S1: Study characteristics: Coaching behavior, antecedents and context, Table S2: Key findings: Coaching behavior, antecedents, and context, Table S3: Study characteristics: Coach–athlete relationship and social support, Table S4: Key findings: Coach–athlete relationship and social support.
