# Supplementary material for: Mental Well-Being or Ill-Being through Coaching in Adult Grassroots Sport: A Systematic Mapping Review
Source: Int J Environ Res Public Health. 2021 Jun 17;18(12):6543. doi: 10.3390/ijerph18126543 (PMC8296415; doi:10.3390/ijerph18126543)
Supplement: Supplementary file 1 [file ijerph-18-06543-s001.zip › ijerph-1223273-supplementary.pdf]

Supplemantary Materials

Table S1: Study characteristics: Coaching behaviour, antecedents and context

**REFERENCE**	**OBJECTIVE (extracted from the article)**	**STUDY DESIGN**	**SAMPLE: SIZE (n) + GENDER (%F/M) + AGE (R/M/SD) + SPORTS + LEVEL** *R= RANGE; M= MEAN; SD= STANDARD DEVIATION*	**COUNTRY OF STUDY**	**INTERVENTION / SETTING / TIME FRAME**
(Aicinena, 2011)	To present examples of hubristic behaviour and the harm that it causes in sport.	Theoretical, hermeneutic	N/A	United States of America	N/A
(Alcaraz et al., 2015)	To test how behavioural regulations are mediated between basic psychological needs and psychological well-being and ill-being in a sample of team-sport coaches.	Quantitative Cross-sectional	N = 302 development coaches<br>18% female; 82% male<br>Rage = 15–53; Mage = 25.97; SD = 8.16<br>Sport: Basketball (58%) and football (42%)<br>Level: regional	Spain	N/A
(Amorose & Anderson-Butcher, 2007)	To explore and better understand the organisational culture of CrossFit	Quantitative Cross-sectional	N = 581; 56,5% female; 45,3% male<br>Sport: a variety of individual and team sports<br>Rage: 13-25; Mage = 17.50; SD = 2.30)	United States of America	N/A
(Appleton & Duda, 2016)	To examine whether a coach-created empowering motivational climate moderated the debilitating effects of a disempowering motivational climate on athletes' health and optimal functioning.	Quantitative Cross-sectional	N = 406 athletes; 32.5% female; 67.5% male<br>Rage = 13-53; Mage =23.1; SD =8.3<br>Level: club (n =254), county (n =50), national (n =102).<br>Sport: a variety of individual (n =61) and team (n =345) sports	United Kingdom	N/A
(Balaguer et al., 2008)	To test a model of the hypothesized motivational sequence among the autonomy support, basic needs, self-determined motivation, self-esteem and life satisfaction on the basis of the self-determination theory.	Quantitative Cross-sectional	N = 301 athletes; 43.2% female; 56.8% male<br>Mage= 24.1; SD = 4.7<br>Sports: a variety of sports.	Spain	N/A
(Bartholomew, Ntoumanis, Ryan, et al., 2011): study 1	To explore (through three studies) the social-environmental conditions that satisfy versus thwart psychological needs and, in turn, affect psychological functioning and well-being or ill-being.	Quantitative Cross-sectional	N = 303 athletes; 100% female<br>Rage = 16-25; Mage = 19.74; SD = 2.19<br>Level: club (n = 51), county (n = 88) regional (n = 57), national (n = 82), or	United Kingdom	N/A

			international (n = 19) level; 6 athletes did not report their competition level.<br>Sport: aesthetic sports such as gymnastics and figure skating (n = 212); or weight-related sports such as light-weight rowing and long-distance running (n = 91).		
(Blanchard et al., 2009)	To test the impact of cohesiveness and coaches' controlling interpersonal style on athletes' perceptions of autonomy, competence and relatedness.	Quantitative Cross-sectional	N = 207 participants; 36,7% female; 59% male; 4,3% did not report their gender.<br>Rage = 16-22; Mage = 18; SD = 1.17<br>Level: inter-cegep (i.e., equivalent of grade 12) basketball league in the Province of Quebec, Canada. Sport: Basketball	Canada	*** N/A
(Borges-Silva et al., 2017)	To analyse the relationships of perceived basic psychological needs, intrinsic motivation and esteem, and life satisfaction in women's fitness training.	Quantitative Cross-sectional	N = 259; 100% female<br>Rage = 18-58; Mage = 34.76; SD = 10.69<br>Sport: Fitness classes (zumba, batuka, aerobic, spinning, step, body pump, etc.)	Spain	*** N/A
(Breske et al., 2017)	To examine the potential for a motivational priming session to buffer the psychophysiological stress response to an ego-involving climate in a physical activity setting.	Quantitative RCT	N = 38; 100% male<br>CG: n = 19<br>IG: n = 19<br>Rage = 18-30; Mage = 20.68; SD = 2.66	United States of America	* (t1) **IG**: exposed to a 10-12 min informational session about Achievement Goal Perspective Theory; **CG**: received a priming session that was neutral in regards to their achievement goals<br>(t2) Juggling sessions<br>** Not reported
(Chinkov & Holt, 2016)	To explore the transfer of life skills among adults who participated in Brazilian jiu-jitsu.	Qualitative Descriptive	N = 16 adults<br>Sport: Brazilian jiu-jitsu	Canada	N/A

			Coaches = 2; 100% male<br>Rage = 27-30<br>Level (belt): brown (1); black (1).<br>Athletes n = 14<br>28,6% female; 71,4% male<br>Rage = 19-54; Mage = 34.6; SD = 10.7<br>Level (belt): white (4); blue (4); purple (2); brown (1); black (1).		
(Denison et al., 2017)	To explore how can athlete empowerment initiatives be anything more than rhetoric within a disciplinary framework that normalizes maximum coach control.	Sociological analysis	N/A	Canada	N/A
(Dumčienė et al., 2015)	To investigate the effects of regular fitness classes on women's satisfaction with their bodies and their sense of coherence.	Quantitative Longitudinal	N = 79<br>100% female<br>Mage = 34.6 ± 3.76	Lithuania	* Fitness classes twice a week in 2013–2014, and not less than half a year group fitness classes<br>* Not reported<br>* 6 months
(Gearity & Metzger, 2017)	To begin to map an understanding of the intersection of sport coaching, mental health, and social identities.<br>To weave together scholarship on microaggressions and the sociology of sport and sport coaching with our stories and interpretations.	Qualitative Post-structural creative analytic	N/A. Three short stories of microaggressions in men's sport coaching and their plausible negative effects on mental health.	United States of America	N/A
(Gearity & Murray, 2011)	To describe the psychological effects of poor coaching reported by collegiate, professional and semi-professional athletes.	Qualitative Existential phenomenolo gy	N = 16 current or former athletes<br>31,2% female; 68,8% male<br>Age: not reported<br>Sport: Baseball, basketball, football, soccer and softball<br>Level: minor leagues (n=2); NFL (n=1); NCAA D.I (n=13)	United States of America	N/A

(Healy et al., 2014)	To clarify mixed results in the literature exploring coach behaviours, basic psychological needs, goal motivation, and well- and ill-being.	Quantitative Cross-sectional & longitudinal	N = 241 athletes<br>34,4% female; 65,6% male<br>Mage = 23.06; SD = 5.45<br>Sport: team sports: hockey = 132; rugby = 16; soccer = 48; volleyball = 23; lacrosse = 11; Gaelic football = 11<br>Level: regional	United Kingdom	N/A<br>* At the beginning of the season: questionnaires (N = 241) + saliva samples to assess physical ill-being (n = 70)<br>* At the end of the season: goal motivation and attainment reported (n = 98).
(Hillier et al., 2019)	To investigate the prevalence, magnitude, methods, and influencers of the practice of rapid weight loss (RWL) in professional and amateur mixed martial arts (MMA) athletes.	Quantitative Cross-sectional	N (for data relating to the method of weight loss and key influencers) = 314 athletes; 8,6% female; 91,4% male<br>Rage = over 18<br>N (for data relating to weight loss magnitude) = 290; 9% female; 91% male<br>Sport: mixed martial arts (MMA)	United Kingdom	N/A
(Hodge & Lonsdale, 2011)	To examine whether the relationships between contextual factors (i.e., autonomy-supportive vs. controlling coaching style) and person factors (i.e., autonomous vs. controlled motivation) outlined in self-determination theory (SDT) were related to prosocial and antisocial behaviours in sport. The authors also investigated moral disengagement as a mediator of these relationships.	Quantitative Cross-sectional	N = 292 athletes; 60% female; 39% male; 1% did not report gender<br>Mage = 19.53; SD = 1.6<br>Level: experienced club-level (n = 77), provincial age-grade (n = 133), national age-group (n = 38), provincial senior (n = 28), and national senior (n = 16)<br>Sport: 39 different team (e.g., netball n = 45; soccer n = 32, field hockey n = 27, basketball n = 14) and individual (e.g., track and field, n = 19; cycling n = 8, swimming n = 7, tennis n = 6) sports	New Zealand	N/A

(Hõs, 2005)	To seek answers of the effects of guided, "age-needs specific," systematic, group aerobic exercise programmes on the self-esteem and self-image of middle-aged women.	Quantitative Quasi-experimental	N = 53<br>100% female<br>Mage = 48.6; SD = 5.1<br>IG: n = 25; Mage = 48.9; SD = 5.6<br>CG: n = 28; Mage = 48.3; SD = 5.2	Hungary	* IG: Professionally guided, age-needs-specific, systematic group aerobic dance exercise programme (low-impact exercises). + CG: No intervention<br>* One hour-long, three times a week for one year.
(Huberty et al., 2008)	To qualitatively examine factors related to physical activity adherence to understand why women continue to participate in long-term exercise after completing a structured exercise program.	Qualitative	n = 19 sedentary faculty and staff at the University of Utah (UTAHFIT participants who attended all of the focus groups)<br>100% female<br>Rage = 26-66 years	USA	* UTAHFIT (U Try Active Habits and Fitness), a PA behaviour change programme.<br>* University of Utah<br>* 12 weeks
(Matosic et al., 2016)	To test a model linking coaches' (n = 59) own reports of narcissistic tendencies with athletes' (n = 493) perceptions of coach controlling behaviours, experiences of need frustration, and attitudes toward doping.	Quantitative Cross-sectional	Athletes: n = 493; 33,5% female; 66,5% male<br>Rage = 16-53; Mage = 21.22; SD = 3.65<br>Coaches n = 59; 18,6% female; 81,4% male<br>Rage = 20-68; Mage = 35.90, SD = 12.71<br>Sport: Rugby, soccer, swimming<br>Level: regional, national, international	United Kingdom	N/A
(Matosic et al., 2017)	(1) To examine the link between a well-researched personality trait, namely narcissism, and two types of coaching interpersonal style, namely autonomy-supportive and controlling styles. (2) To test the mediating roles of dominance and empathic concern in explaining the relations between narcissism and the two coaching interpersonal styles.	Quantitative Cross-sectional	N = 211 professionally qualified coaches<br>15,6% female; 84,4% male<br>Rage = 18-81; Mage = 38.30; SD = 14.16<br>Sport: a variety (n = 28) of sports (e.g., football, rugby, cricket, swimming, athletics, tennis).	United Kingdom	N/A

(Mickelsson, 2020)	To explore how the currently underexplored martial art disciplines may contribute to sociopsychological development among young people. In addition, it was investigated whether individuals who are predisposed to different traits may favour one sport over the other.	Quantitative Longitudinal	N = 145; 125 males, 20 females<br>Rage = 15-24; Mage = 20.23; SD = 2.43<br>Sport: local martial arts<br>MMA group: n = 79<br>BJJ group: n = 66	Sweden	Training Mixed Martial Arts (MMA) or Brazilian Jiu-Jitsu (BJJ)<br>5 months
(Norris et al., 2017)	To conduct a systematic review of literature on stressors, coping, and well-being among sports coaches.	Systematic review	38 studies that were conducted with 4188 sports coaches. This sample consisted of 19 qualitative, 17 quantitative, and two mixed methods studies	United Kingdom	N/A
(Schüler et al., 2016)	To examine whether implicit or explicit autonomy dispositions moderate the relationship between felt autonomy and well-being.	Quantitative Cross-sectional	**Study 1:**<br>N = 187 undergraduate students<br>87,2% female; 12,8% male<br>Mage = 21.6; SD = 5.76<br>**Study 2**:<br>N = 127 physically inactive persons<br>64,6% female; 35,4% male<br>Rage = 15-64; Mage = 29.9; SD = 13.9	Switzerland / United States of America	N/A
(Smith et al., 2007)	To examine the motivational processes underlying goal striving in sport as well as the role of perceived coach autonomy support in the goal process.	Quantitative Cross-sectional	N = 210 regularly training athletes<br>49,1% female; 49,5% male; 1,4% unspecified<br>Rage =18-37; Mage = 21.02; SD = 2.88<br>Level: locally to international<br>Sport: a variety of individual and team sports (e.g., football, netball, and badminton.	United Kingdom	N/A
(Smith et al., 2010)	To investigate the influence of coach behaviours and implementation intentions on goal striving in sport:<br>(1) to further examine the role of goal motives in sport and specifically to address some of their antecedents and consequences using a prospective design. (2) To investigate the interactions (or lack thereof) between autonomous and	Quantitative Prospective	n final = 108<br>**Time 1**:<br>N = 189 regularly training athletes<br>50,3% female; 46% male; 3,7% unspecified<br>**Time 2** (8 weeks later):<br>n = 108; 41,7% female; 58,3% male	United Kingdom	N/A<br>8 weeks + online follow up

	controlled goal motives and implementation intentions in predicting goal progress and relative well-being. (3) to examine whether the motives underlying implementation intentions might be important in understanding how goal motives predict progress and well-being.		Rage: 18-67; Mage = 23.97; SD = 9.77 Sport: variety of individual (i.e., badminton, triathlon, track athletics) and team sports (i.e., rowing, cricket, netball, basketball, soccer, field hockey). Level: local (17.6%), regional (15.7%), national (19.4%), international (6.5%), university (39.8%).		
(Soulliard et al., 2019)	(1) To examine differences in positive body image, specifically body appreciation and functionality appreciation, between student athletes and non-athletes. (2) To examine the relationships between positive body image and other sport-related variables	Quantitative Cross-sectional	N = 254 undergraduate students from a NCAA Division I private university: Student athletes: n = 79; 67,1% female; 32,9% male; Rage = + 18; Mage = 19.79; SD = 1.13; Sports: baseball (n = 8), basketball (n = 2), cheerleading/dance (n = 7), cross country/track and field (n = 24), field hockey (n = 3), soccer (n = 10), softball (n = 6), swimming/diving (n = 11), tennis (n = 5), and volleyball (n = 3). Non-athletes n = 175; 72,6% female; 26,9% male; Rage = + 18; Mage = 19.38; SD = 1.81	United States of America	N/A
(Stebbings et al., 2011)	To test a model of potential antecedents of perceived coach autonomy supportive and controlling behaviours using the SDT framework.	Quantitative Cross-sectional	N = 443 coaches : 29,3% female; 70,7% male Rage = 18-75; Mage = 41.06; SD = 14.24 Level: recreational (n = 52), club (n = 174), regional (n = 73), national (n = 80), international / professional (n = 64).	United Kingdom	N/A
(Stebbings et al., 2012)	To test a BPNT-based model of potential antecedents of perceived coach interpersonal behaviour	Quantitative Cross-sectional	n = 418 coaches; 26,8% female; 73,2% male Rage = 18-78; Mage = 43.68; SD = 14.41 Level: recreational (n = 66), club (n = 187), county (n = 86), national (n = 51), international/professional (n = 28)	United Kingdom	N/A
(Stebbings et al., 2015)	To explore sports coaches' psychological well-being (positive affect and integration of coaching with one's	Quantitative Longitudinal	N = 195 coaches; 21% female; 79% male Rage = 18-75; Mage = 46.24; SD = 13.26	United Kingdom	** N/A * Measures at three

	sense of self) and ill-being (negative affect and devaluation of coaching) as predictors of their perceived autonomy supportive and controlling interpersonal styles towards individuals under their instruction.		n who completed all 3 measures = 119<br>n who completed 2 measures = 76<br>Level: recreational (n = 20), club (n = 81), regional (n = 37), national (n = 41), international/ professional (n = 16).		time points across an eleven-month period, with time points two and three approximately five and eleven months after time point one, respectively.
(Torregrosa et al., 2014)	To analyse the relationship of the controlling coach behaviour, perceived autonomous support practitioners about the coach and the psychological mediators with the life satisfaction in healthy exercise practitioners.	Quantitative Cross-sectional	N = 104 non-competitive exercise practitioners; 100% male<br>Rage: 18-70; Mage = 28.38; SD = 11.66<br>Sport: wellness	Spain	N/A
(Wayment & McDonald, 2017)	To examine a novel personal fitness training program that combines personal training principles in a small-group training environment.	Mixed Methods Cross-sectional	N = 98; 65,3% female; 32,7% male<br>Rage = 19-78; Mage = 46.52; SD = 14.15<br>Sport: Fitness	United States of America	N/A

Table S2: Key findings: Coaching behaviour, antecedents and context

REFERENCE	CATEGORIES ANALISED AND MEASUREMENT INSTRUMENTS	KEY FINDINGS REPORTED (extracted from the article)
(Aicinena, 2011)	* Pride and hubristic behaviour	Examples of hubristic behaviour and the harm that it causes in sport are presented.
(Alcaraz et al., 2015)	* C**oaches' Basic Psychological Need (BPN) satisfaction**:<br>- **Need for autonomy**: four items from the ***Standage, Duda, and Ntoumanis's research (2005)***.<br>- **Need for competence:** four items from the ***Perceived Competence scale of the Intrinsic Motivation Inventory*** (McAuley, Duncan, & Tammen, 1989).<br>- **Need for relatedness:** four items from the ***Acceptation scale of the E´chelle du Sentiment d'Appartenance Sociale*** (Richer & Vallerand, 1998).<br>* To what **degree coaches' BPN were thwarted**: ***Psychological Need Thwarting Scale*** (Bartholomew, Ntoumanis, Ryan, & Thøgersen-Ntoumani, 2011).<br>* **Behavioral Regulations**: **adaptation of the Behavioral Regulation in Sport Questionnaire (BRSQ**; Lonsdale, Hodge, & Rose, 2008) to the coaching context.<br>* **Psychological well-being**:<br>- **Coaches' positive affect**: five items from the ***Positive and Negative Affect Scale (PANAS***; Watson, Tellegen, & Clark, 1988).<br>- **Coaches' subjective vitality**: five items from the ***Subjective Vitality Scale*** (Ryan & Frederick, 1997).<br>* **Coaches' ill-being**: negative affect and perceived stress.<br>- **Negative affect:** five items from the ***PANAS***.<br>- **Coaches' perceptions of stress**: short form of the ***Perceived Stress Scale (PSS-10***; see Cohen & Janicki-Deverts, 2012).	The results provided support for the partial mediation model. Coaches' motivation mediated the relationships from both relatedness need satisfaction and basic psychological needs thwarting for coaches' well-being. In contrast, relationships between basic psychological needs satisfaction and thwarting and ill-being were only predicted by direct effects. Results highlight that 3 conditions seem necessary for coaches to experience psychological well-being in their teams: basic psychological needs satisfaction, especially relatedness; lack of basic psychological needs thwarting; and self-determined motivation.
(Amorose & Anderson-Butcher, 2007)	* Athletes' perception of the **autonomy-supportive coaching**: ***short version of the Sport Climate Questionnaire (SCC)***.<br>* **Fundamental human needs (athletes' perceptions of competence,**	Participant accounts revealed that the artefacts of the organisational culture included the rugged, industrial appearance of the gym (visual structures and processes) and the social nature of members' interactions prior

	**autonomy, and relatedness**): same ***measures employed by Hollembeak and Amorose (2005)***.<br>* **Motivational orientation (athletes' motivation for participating in their sport)**: ***The Sport Motivation Scale*** (Pelletier et al., 1995).	to/following each workout (observable behaviour). Espoused beliefs and values identified included pride in the gym and their workouts, inclusivity, and a strong sense of community that extended beyond the gym. A shared underlying assumption was the common goal of improving their health and well-being.
(Appleton & Duda, 2016)	* Participants' perceptions of coach-created **Empowering and disempowering motivational climates:** *EDMCQ-C* (Appleton, Ntoumanis, Quested, Viladrich, & Duda, 2016).<br>- The *empowering* climate items measure **task-involving**, **autonomy-supportive** and **socially-supportive coaching**.<br>- The *disempowering* climate items measure **ego- involving** and **controlling climate dimensions**.<br>* **Enjoyment:** The *enjoyment subscale from the Intrinsic Motivation Inventory* (McAuley, Duncan, & Tammen, 1989)<br>* **Athlete burnout:** The 15-item *Athlete Burnout Questionnaire (ABQ*; Raedeke & Smith, 2009):<br>- **participants' self-reported reduced sense of athletic accomplishment**<br>- **perceived emotional and physical exhaustion**<br>**- sport devaluation**<br>* **Global self-esteem:** A *5-item global self-esteem measure* was obtained *from the Short Version of the Physical Self Description Questionnaire* (Marsh, Martin, & Jackson, 2010)<br>* Participants' experiences of **Symptoms of physical ill-health:** *Physical Symptom Checklist* (Emmons, 1991).	The interaction between disempowering and empowering climate dimensions was significant and predicted 1% unique variance in 3 outcome variables (i.e., enjoyment, reduced accomplishment, and physical symptoms). The Johnson-Neyman technique was employed to plot and probe the significant interactions, which revealed moderately strong to strong values of an empowering climate tempered the significant relationship between a disempowering climate and the three outcome variables. The findings from this study have implications for coach education and suggest programmes that train coaches to understand how to create empowering climates and avoid (or dramatically reduce) disempowering climates are warranted.
(Balaguer et al., 2008)	* **Apoyo a la Autonomía:** ***versión castellana*** (Balaguer, Castillo, Duda, Álvarez, y Díaz, 2004; Balaguer, Castillo y Duda, 2008), del Cuestionario de Clima en el Deporte (***Sport Climate Questionnaire***, n. d.). Tiene sus ***orígenes en el Health-Care-Climate Questionnaire (HCCQ***, Williams, Grow, Freedman, Ryan y Deci, 1996).<br>* **Competencia:** ***Escala de Competencia Percibida del Cuestionario de Motivación Intrínseca (McAuley, Duncan, y Tammen***, 1989).	The robust method structural equation modelling analysis using maximum likelihood (LISREL 8.54) showed that athletes' perceptions of autonomy support from coaches predicted their satisfaction of the needs for autonomy and relatedness. These needs, along with perceived competence, predicted self-determined motivation, which in turn corresponded to greater self-esteem and life

	* **Autonomía:** ***Escala de Autonomía Percibida en el Deporte elaborada por Reinboth y Duda*** (2006).<br>* **Relación:** ***subescala de Aceptación de la Escala de Necesidad de Relación de Richer y Vallerand*** (1998).<br>* **Motivación auto-determinada:** ***versión castellana*** (Balaguer, Castillo y Duda, 2003, 2007) de la ***Escala de Motivación Deportiva (SMS***; Pelletier et al., 1995)<br>* **Autoestima:** ***subescala de Auto-Valía del Cuestionario de Autodescripción (SDQ-III***; Marsh, Richards, Johnson, Roche y Tremayne, 1994).<br>* **Satisfacción con la vida:** ***versión castellana*** (Atienza, Pons, Balaguer y García-Merita, 2000) de la ***Escala de Satisfacción con la Vida (SWLS***; Diener, Emmons, Larsen y Griffin, 1985).	satisfaction.
(Bartholomew, Ntoumanis, Ryan, et al., 2011): study 1	**Study 1:**<br>* Athletes' perceptions of their coach's **Autonomy-supportive behaviors**: **six items taken from the *Health-Care Climate Questionnaire (HCCQ***; Williams, Grow, Freeman, Ryan, & Deci, 1996) and **modified for their use in sport.**<br>* Athletes' perceptions of their coach's **Controlling behaviors**: **15-item *Controlling Coach Behaviors Scale (CCBS***; Bartholomew et al., 2010): **controlling use of rewards**, **negative conditional regard**, **intimidation**, and **excessive personal control**.<br>* **Need satisfaction**:<br>(1) **satisfaction of the need for autonomy**: five items collated by ***Standage, Duda, and Ntoumanis (2003)***.<br>(2) **Satisfaction of the need for competence: five items from the *competence subscale of the Intrinsic Motivation Inventory (IMI***; McAuley, Duncan, & Tammen, 1989).<br>(3) **Satisfaction of the need for relatedness: 5-item *acceptance subscale of the Need for Relatedness Scale (NRS-10***; Richer & Vallerand, 1998).<br>* **Need thwarting**: **12-item *Psychological Need Thwarting Scale (PNTS***; Bartholomew et al., 2011).<br>* **Disordered eating**: ***Questionnaire for Eating Disorder Diagnoses (Q-EDD***; Mintz, O'Halloran, Mulholland, & Schneider, 1997).	In cross-sectional Studiy 1, structural equation modelling analyses supported latent factor models in which need satisfaction was predicted by athletes' perceptions of autonomy support, and need thwarting was better predicted by coach control. Athletes' perceptions of need satisfaction predicted positive outcomes associated with sport participation (vitality and positive affect), whereas need thwarting more consistently predicted maladaptive outcomes (disordered eating, burnout, depression, negative affect, and physical symptoms). In addition, athletes' perceptions of psychological need thwarting were significantly associated with perturbed physiological arousal (elevated levels of secretory immunoglobulin A) prior to training. The final study involved the completion of a diary and supported the relations observed in the cross-sectional studies at a daily level.

	* **Vitality (athletes' feelings of positive energy)**: ***five-item version of the Subjective Vitality Scale (SVS***; Ryan & Frederick, 1997)<br>* **Depression**: 7-item ***depression subscale of the Depression Anxiety Stress Scale (DASS***; Lovibond & Lovibond, 1995).	
(Blanchard et al., 2009)	* Coach's controlling interpersonal style**:** (Based on Grolnick, Ryan &Deci, 1991) three items assesses the **extent to which athletes perceive their coach as controlling**<br>* **Cohesiveness** (extent to which the team is united in reaching the same goal)**:** ***group integration-task subscale from the Group Environment Questionnaire (GEQ***: Carron, Widmeyer, & Brawley, 1985).<br>* Perception of need satisfaction**:** 8 items assess the **perceptions of autonomy, competence and relatedness**.<br>* ***The sport motivation scale (SMS;*** adapted from Pelletier et al., 1995 and Vallerand & O'Connor, 1991).<br>* **Subjective well-being** in sports: *two subscales*:<br>**1) athletes' positive emotions** experienced when playing basketball<br>**2)** their **degree of satisfaction** with their sport	Perceptions of cohesiveness positively predicted the satisfaction of the basic needs. Perceptions of coaches'' controlling interpersonal style negatively impacted feelings of autonomy. In turn, psychological needs predicted self-determination in sports ensuing greater sport satisfaction and positive emotions in sports. Tests of indirect effects also supported the mediating role of psychological needs and self-determination.
(Borges-Silva et al., 2017)	* **Percepción de satisfacción de las necesidades psicológicas básicas** en contextos de ejercicio físico. ***Psychological Need Satisfaction in Exercise Scale (PNSE)*** de Wilson, Rogers, Rodgers, y Wild (2006) ***validada al contexto español*** por Moreno-Murcia, Marzo, Martínez-Galindo, y Conte (2011): **competencia**, **autonomía** y **relación con los demás**.<br>* **Motivación intrínseca**. ***subescala del Behavioral Regulation in Sport Questionnaire (BRSQ)*** de Lonsdale, Hodge, y Rose (2008), ***validado al contexto español*** por Moreno-Murcia et al. (2011) y adaptado al contexto de ejercicio físico.<br>* **Autoestima**. ***dimensión autoestima perteneciente al cuestionario de medida del autoconcepto físico de Moreno y Cervelló (2005), tomado del original Physical Self-Perception Profile (PSPP)*** de Fox y Corbin (1989).<br>* **Satisfacción con la vida**. ***cuestionario Escala de Satisfacción para la Vida (ESDV-5)*** de Vallerand, Blais, Briére, y Pelletier (1989) ***validada al contexto español.***	Using structural equation analysis, perceived basic psychological needs positively predicted intrinsic motivation, positively influencing esteem and satisfaction with lifetime. The predictive model exposed the importance of promoting satisfaction of autonomy, competence and social relationships for improved self-esteem. Consequently, greater satisfaction with the women's lives towards obtaining positive effects from practicing fitness classes were found.

(Breske et al., 2017)	**Psychophysiological stress responses (e.g., cortisol)**<br>Cortisol was measured at five time points throughout the study via ***saliva samples***.<br>***self-reported anxiety and self-confidence (CSAI-2)***.	Results showed a marked increase in cortisol (as measured by percent change from baseline) in the control group, but not the experimental group. Psychological responses were stable across groups. Conclusion Providing athletes and exercisers with nothing more than basic information on AGPT can reduce their physiological markers of psychosocial stress in ego-involving climates. Such education may be a beneficial practice for coaches, physical educators, and trainers.
(Chinkov & Holt, 2016)	*** Perceived Influence of BJJ on Participants' Lives**<br>*** Reported Life Skills:**<br>**- Respect for others**<br>**- Perseverance**<br>**- Self-confidence**<br>**- Healthy habits**<br>*** Ways Life Skills Were Acquired**<br>*** Peer Support**<br>***Individual semistructured interviews***. Thematic analysis.	Participants thought their involvement in Brazilian jiu-jitsu had changed their lives. These changes occurred via the acquisition of four life skills reflecting values and characteristics of the sport: respect for others, perseverance, self-confidence, and healthy habits. Head instructors and peer support facilitated the acquisition of life skills. Combined, the values of the sport, instructors, and peers created an atmosphere for learning life skills implicitly.
(Denison et al., 2017)	* Sports' disciplinary legacy	Coaching with greater consideration for athletes' unique qualities and developmental differences needs to entail coaching in a less disciplinary way and with an awareness and appreciation of the many unseen effects that disciplinary power can have on coaches' practices and athletes' bodies.
(Dumčienė et al., 2015)	* ***Body Shape Questionnaire*** (Cooper, Taylor, Cooper, & Fairburn, 1986): women's dissatisfaction with their body shapes.<br>* **sense of coherence level**: ***Antonovsky (1987) 13 items scale***: three subscales: **comprehensibility** (cognitive component), **meaningfulness** (the motivational component) and **manageability** (the behavioural component).	After six months of regular fitness classes women's satisfaction of their body and their sense coherence level improved significantly. Relations between body shape dissatisfaction and sense of coherence, body shape dissatisfaction and manageability, comprehensibility and manageability were found. Conclusions. Systematic fitness training positively affects women's satisfaction

		with their body. Understanding of the capability of controlling their body shape was improved. There was a significant relationship between dissatisfaction with their body shape and sense of coherence. The effect of physical activity improved the values of all three components of the sense of coherence construct. The values of comprehensibility, meaningfulness and manageability indicators show that respondents may already have a better control of their sense of coherence.
(Gearity & Metzger, 2017)	* Microaggressions: Race, gender identity, sexual orientation, and religious microaggressions<br>* Microaffirmations<br>* Implications of microaggressions for coaching practice<br>* Intersection of sport coaching, mental health, and social identities: ***stories and interpretations***	Microaggressions are exercised as disciplinary power to control athletes' bodies. A discursive understanding of power-knowledge produces coach and athlete identities and practices, and some plausible mental health effects resulting from these interactions.
(Gearity & Murray, 2011)	***experiences of poor coaching***:<br>(1) poor teaching by the coach<br>(2) uncaring<br>(3) unfair<br>(4) inhibiting athlete's mental skills<br>(5) athlete coping.	The five themes derived from athletes' reports were: poor teaching by the coach, uncaring, unfair, inhibiting athlete's mental skills, and athlete coping. Two of these themes, inhibiting athlete's mental skills and coping, are closely connected to psychological constructs, and are presented in this paper. The theme of inhibiting athlete's mental skills was made up of athletes' descriptions of poor coaches as being distracting, engendering self-doubt, demotivating, and dividing the team. The theme of athlete coping describes how athletes responded to being poorly coached. Conclusions: Researchers conclude that the two themes, inhibiting athlete's mental skills and athlete coping, are related to several constructs in sport psychology literature such as motivation, self-efficacy, focus and concentration, team cohesion, and stress and coping. Instruction on coping skills is warranted for athletes dealing with poor coaching.

(Healy et al., 2014)	* **Perceptions of coach behaviours**:<br>- **perception of coach autonomy support**: ***adapted items from the Health-care climate questionnaire*** (Williams, Grow, Freedman, Ryan, & Deci, 1996).<br>- **Perception of coach controlling behaviour:** ***Controlling Coach Behaviors Scale*** (Bartholomew et al., 2010).<br>* **Basic Psychological Needs Satisfaction and Thwarting**: ***Basic Needs Satisfaction in Sport Scale (BNSSS***; Ng, Lonsdale, & Hodge, 2011) & ***Psychological Need Thwarting Scale (PNTS***; Bartholomew et al., 2011b).<br>* **Goal-related variables**: Athletes identified their **most important personal goal** for the season and rated the extent that athletes were striving with **extrinsic**, **introjected**, **identified** and **intrinsic motives**.<br>* **Well-being and Ill-being**:<br>- ***Subjective Vitality Scale (SVS***; Ryan & Frederick, 1997): **psychological well-being**.<br>- **psychological ill-being**: ***Athlete Burnout Questionnaire (ABQ***; Raedeke & Smith, 2001): three subscales: **Reduced sense of accomplishment**, **Devaluation** and **Emotional/Physical exhaustion**<br>* **Physical ill-being symptoms**: ***Physical Symptoms Checklist (Emmons, 1991)***.<br>- **S-IgA** was measured using ***saliva samples***	Structural equation modelling demonstrated that coach behaviours were related to needs satisfaction and thwarting, which were related to autonomous and controlled goal motives respectively. Autonomous motives were related to well- and ill-being; controlled motives were only related to ill-being. Over time, only end-of-season autonomous goal motives were related to goal attainment. The findings provide an insight into how coaches can facilitate optimum goal striving and well-being in their athletes.
(Hillier et al., 2019)	* Validated ***Rapid Weight Loss Questionnaire (RWLQ)*** was adapted for the current study to ensure appropriateness to MMA. The questions covered the level and frequency of competition, training, athletic achievements, weight history, diet and RWL.	Sex-specific data were analysed, and subgroup comparisons were made between athletes competing at professional and amateur levels. Most athletes purposefully reduced body weight for competition (men: 97.2%; women: 100%). The magnitude of RWL in 1 week prior to weigh-in was significantly greater for professional athletes compared with those competing at amateur level (men: 5.9% vs. 4.2%; women: 5.0% vs. 2.1% of body weight; $p < .05$). In the 24 hr. preceding weigh-in, the magnitude of RWL was greater at professional than amateur level in men (3.7% vs. 2.5% of body weight; $p < .05$). Most athletes "always" or "sometimes" used water loading (72.9%), restricting fluid intake (71.3%), and sweat

		suits (55.4%) for RWL. Coaches were cited as the primary source of influence on RWL practices (men: 29.3%; women: 48.1%). There is a high reported prevalence of RWL in MMA, at professional and amateur levels. The findings call for urgent action from MMA organizations to safeguard the health and well-being of athletes competing in this sport.
(Hodge & Lonsdale, 2011)	* **Athletes' perceptions of Autonomy-Supportive and Controlling Coaching Styles**:<br>- ***14 items adapted from the Health Care Climate Questionnaire*** (Williams, Cox, Kouides, & Deci, 1999): **autonomy-supportive coaching style**<br>- ***4 items from the College-Student Scale*** (Grolnick, Ryan, & Deci, 1991): **controlling coaching style** (e.g., "My coach insists that I do things his/her way") in competitive sport.<br>* ***Behavioral Regulation in Sport Questionnaire-6 (BRSQ-6)*** (Lonsdale, Hodge, & Rose, 2008): six types of motivational regulation as specified in SDT. subscales to measure **intrinsic motivation** (IM), **integrated regulation** (IG), **identified regulation** (ID), **introjected regulation** (IJ), **external regulation** (EX), and **amotivation** (AM).<br>* ***Moral Disengagement in Sport Scale–Short (MDSS-S)*** (Boardley & Kavussanu, 2008): **athletes' overall sport moral disengagement**.<br>* Athletes' ***Prosocial and Antisocial Behavior in Sport Scale (PABSS)*** (Kavussanu & Boardley, 2009): four subscales: (i) **prosocial behaviour toward teammates**, (ii) **prosocial behaviour toward opponents**, (iii) **antisocial behaviour toward teammates**, and (iv) **antisocial behaviour toward opponents**.	Results indicated that an autonomy-supportive coaching style was associated with prosocial behaviour toward teammates; this relationship was mediated by autonomous motivation. Controlled motivation was associated with antisocial behaviour toward teammates and antisocial behaviour toward opponents, and these two relationships were mediated by moral disengagement. The results provide support for research investigating the effect of autonomy-supportive coaching interventions on athletes' prosocial and antisocial behaviour.
(Hõs, 2005)	* ***Rosenberg Self-esteem Scale (1965)***: **self-esteem**<br>* ***Tennessee Self-image Test*** (Dévai & Sipos, 1986): total self-image (TS) scale: **body image (BI), moral self-image (MS), individual self-image (IS), family self-image (FS), social self-image (SS).**	The results of the study showed significant improvements in body image for those middle-aged women who participated in the one- year long aerobic dance exercise programme, while the body images of the control group remained the same. This shows the important intervening role of satisfaction with body image of middle-aged

		women between systematic aerobic dancing and self-esteem. The one year long systematic aerobic dance programme had a positive effect on self-image, self-esteem, physical condition, and an evaluation of the environment of middle-aged women. Conclusions: an improved body image can positively influence and stabilize self-esteem. Furthermore, the improved self-esteem and self- image can contribute to improvements of quality in the lives of middle-aged women and it may compensate for the negative effects of the menopausal period.
(Huberty et al., 2008)	* ***Demographic and health history questionnaire***<br>* ***Modifiable Activity Questionnaire (MAQ)***: **adherence** classification.<br><br>Data were collected from ***focus groups, interviews***, and ***e-mails***, and analysis used grounded theory.	The central category related to physical activity adherence was self-worth. Motivation, activity enjoyment, priorities, body image, ability to access support, and self-regulation skills had an impact on the self-worth of non-adherers and adherers. Women must value themselves enough to continue to participate in physical activity once they start. Exercise and fitness professionals are encouraged to use strategies to increase self-worth and long-term adherence to physical activity. Some recommended strategies include (a) increasing motivation and enjoyment relative to activity, (b) making activity a high priority in a woman's 4/c, (c) improving or deemphasizing body image, (d) increasing a woman's ability to access support, and (e)facilitating the use of self-regulation strategies.

(Matosic et al., 2016)	* **Coach narcissism**: ***40-item Narcissistic Personality Inventory (NPI***; Raskin & Terry, 1988).<br>* **Coach dominance**: ***11-item International Personality Item Pool (IPIP***: Goldberg et al., 2006), based on the California Personality Inventory (CPI; Wink & Gough, 1990)<br>* **coach empathic concern**: ***7-item empathic concern subscale of the Interpersonal Reactivity Scale (IRI;*** Davis, 1983).<br>* **Athletes' perceptions of their coach's controlling behaviors**: ***15-item Controlling Coach Behaviors Scale (CCBS***; Bartholomew, Ntoumanis, & Thøgersen-Ntoumani, 2010).<br>* **Need frustration**: ***12-item Psychological Need Thwarting Scale (PNTS***; Bartholomew et al., 2011b). three subscales corresponding to athletes' autonomy, competence, and relatedness needs.<br>* **Attitudes toward doping**: ***5-item modified version of the Performance Enhancement Attitude Scale (PEAS***; Petróczi & Aidman, 2009) by Gucciardi, Jalleh, and Donovan (2011).	Multilevel path analysis revealed that coach narcissism was directly and positively associated with athletes' perceptions of controlling behaviours and was indirectly and positively associated with athletes' reports of needs frustration. In addition, athletes' perceptions of coach behaviours were positively associated-directly and indirectly-with attitudes toward doping. The findings advance understanding of controlling coach behaviours, their potential antecedents, and their associations with athletes' attitudes toward doping.
(Matosic et al., 2017)	* **autonomy-supportive and controlling coach behaviours**: ***12 vignettes*** which corresponded to the 12 most important characteristics of narcissism: hypersensitivity to criticism, authority, self-sufficiency, superiority, exhibitionism, exploitativeness, entitlement, feelings of inferiority, lack of empathy, amorality, arrogance, and grandiosity.<br>* **narcissism: *40-item and forced-choice Narcissistic Personality Inventory (NPI***; Raskin & Terry, 1988).<br>* **dominance: *11-item International Personality Item Pool Dominance Scale*** (Goldberg et al., 2006), based on the California Personality Inventory (Wink & Gough, 1990).<br>* **Empathic concern: *7-item Empathic Concern Subscale of the Interpersonal Reactivity Scale*** (Davis, 1980).	Regression analyses revealed a positive direct relation between narcissism and controlling coach behaviours. Furthermore, empathy (but not dominance) mediated the positive and negative indirect effects of narcissism on controlling and autonomy-supported interpersonal styles, respectively.
(Mickelsson, 2020)	* **Aggression: *Buss-Perry Aggression Questionnaire (BPAQ***; Buss & Perry, 1992). four subscales: **physical aggression**, **verbal aggression**, **hostility** and **anger**.<br>* **Pro-social behaviour: *Prosocialness Scale for Adults (PSA***; Caprara, Steca,	The results show that both groups displayed increased self-control and pro-social behaviour; however, MMA practitioners also reported increased aggressiveness, whereas BJJ practitioners experienced a decline in

	Zelli, & Capanna, 2005).<br>* **Self-control:** ***Self-Control Scale*** (***SCS***; Alvarez-Rivera & Talbot, 2010).<br>* **Criminal frequency**. ***Total Delinquent Acts Measure (TDAM***; Elliott, Huizinga, & Ageton, 1985).	aggression. Accordingly, individuals who trained in MMA displayed substantially higher pre-existing aggression levels than the BJJ practitioners. The current results further corroborate research suggesting that modern martial arts and MMA may not be suitable for at-risk youth to practice, whereas traditional martial arts and sports with a healthy philosophical foundation may be effective in reducing antisocial behaviour while enhancing socially desirable behaviour among young people.
(Norris et al., 2017)	N/A (systematic review)	The findings demonstrate that coaches experience a variety of stressors relating to their performance and that of the athletes they work with in addition to organizational, contextual, interpersonal, and intrapersonal stressors. The findings also highlight that coaches use a variety of coping strategies (e.g., problem solving, social support, escaping the stressful environment) to reduce the negative outcomes of stress. Five studies that were included in this review focused on coaches' well-being and found that basic psychological needs satisfaction, lack of basic psychological needs thwarting, and self-determined motivation are needed for coaches to be psychologically well.
(Schüler et al., 2016)	* ***Picture Story Exercise***: **implicit motive dispositions** (McClelland et al., 1989; Murray, 1943).<br>* ***Operant Motive Test (OMT)***. **implicit autonomy and power and achievement motives from the OMT**. Specifically, the OMT freedom categories were used to score for the **implicit autonomy disposition**. The OMT's power categories and achievement categories were used to assess the **implicit power and achievement motive**, respectively.<br>* **Autonomy Satisfaction**: Autonomy subscale of the ***Basic Psychological Needs Scale (Gagné, 2003; Kashdan, Julian, Merritt, & Uswatte, 2006)***.<br>* **Flow experience**: ***Flow Short Scale (FSS***; Rheinberg, Vollmeyer, & Engeser,	Study 1 showed that individuals with a strong implicit autonomy (but not power or achievement) motive disposition derived more flow experience from felt autonomy than individuals with a weak implicit autonomy disposition. Study 2 revealed that perceived autonomy support from sports coaches, which was experimentally induced with a vignette method, leads to autonomy satisfaction, leading in turn to positive effects on well-being. This indirect effect held at high and average but not low implicit autonomy disposition. The

	2003): two subscales: **Flow-Absorption** and **Flow-Automaticity**.	results indicate that the degree to which people benefit from autonomy need satisfaction depends on their implicit disposition toward autonomy.
(Smith et al., 2007)	* **Personal Goals**: ***idiographic goal methodology advocated within self-concordance research (Sheldon, 2002).*** to nominate four goals that they were currently pursuing.<br>* **Need Satisfaction**: Satisfaction of the basic psychological needs for autonomy, competence, and relatedness: ***six autonomy items from Standage, Duda, and Ntoumanis (2005), six items from the perceived competence subscale of the Intrinsic Motivation Inventory*** (McAuley, Duncan, & Tammen, 1989), ***and five items from the acceptance subscale of the Need for Relatedness Scale*** (Richer & Vallerand, 1998), respectively.<br>* **Psychological well-being: *20-item Positive and Negative Affect Schedule*** (Watson, Tellegen, & Clark, 1988), ***five-item Satisfaction With Life Scale*** (Diener, Emmons, Larsen, & Griffin, 1985), and ***five-item emotional/physical exhaustion subscale of the Athlete Burnout Measure*** (Raedeke & Smith, 2001).<br>* **Perceptions of coach autonomy support: *seven items taken from the Health-Care Climate Questionnaire*** (Williams, Grow, Freedman, Ryan, & Deci, 1996) modified for sport.	Structural equation modelling with a sample of 210 British athletes showed that autonomous goal motives positively predicted effort, which, in turn, predicted goal attainment. Goal attainment was positively linked to need satisfaction, which, in turn, predicted psychological well-being. Effort and need satisfaction were found to mediate the associations between autonomous motives and goal attainment and between attainment and well-being, respectively. Controlled motives negatively predicted well-being, and coach autonomy support positively predicted both autonomous motives and need satisfaction. Associations of autonomous motives with effort were not reducible to goal difficulty, goal specificity, or goal efficacy. These findings support the self-concordance model as a framework for further research on goal setting in sport.
(Smith et al., 2010)	* **Goal-Related Measures**: ***self-generate*** a salient **personal sports goal** for the entire sports season.<br>* **Goal Motives** and **Goal Difficulty**: using the idiographic goal methodology advocated by Sheldon and Elliot (1999). ***participants rated*** **the extent to which they were pursuing their goals in terms of four reasons reflecting intrinsic**, **identified**, **introjected**, and **external regulations**.<br>* **Implementation Intentions**: participants' personal use of implementation intentions, that means, planning the when, where, and how of goal striving, in addition to "if-then" plans for goal-related behaviours.<br>* **Perceptions of Coach Behaviours**:<br>**- Participants' perceptions of coach autonomy support**<br>**- Perceptions of coaches' controlling behaviours:** The ***scale*** included the use of controlling statements and conditional regard, the provision of punishments	Structural equation modelling analysis with a sample of 108 athletes revealed coach behaviours as predictors of goal motives, which in turn predicted psychological well-being after 8 weeks. Supplementary regression analyses showed no interaction between autonomous goal motives and implementation intentions; however, a synergistic effect was identified for controlled goal motives such that controlled motives furnished with implementation intentions resulted in lower well-being than controlled motives alone. In further analyses, the motives underlying an implementation intention were found to mediate the paths from goal motives to well-being. The findings are discussed in terms of the roles played by goal motives,

	and rewards without competence information, the demonstration of overt physical control, and the prompting of ego-involving motives.<br>* **Psychological Well-Being:**<br>- ***Positive and Negative Affect Schedule*** (Watson, Tellegen, & Clark, 1988)<br>- ***Satisfaction With Life Scale*** (Diener, Emmons, Larsen, & Griffin, 1985)<br>- ***Emotional/physical exhaustion subscale of the Athlete Burnout Measure*** (Raedeke & Smith, 2001).	implementation intentions, and implementation intention motives during goal striving.
(Soulliard et al., 2019)	* ***Body Appreciation Scale-2 (BAS-2)*** (Tylka & Wood-Barcalow, 2015a).<br>* ***Functionality Appreciation Scale (FAS)*** (Alleva et al., 2017).<br>* ***Dispositional Flow Scale – 2 (DFS-2)*** (Jackson, Martin, & Eklund, 2008): **general tendency to experience flow characteristics during physical activity**.<br>* ***Trait Sport-Confidence Inventory (TSCI)*** (Vealey, 1986): **how confident athletes generally feel when competing in sport**.<br>* ***Subjective Performance Questionnaire (SPQ)*** (new scale developed by the authors for the purposes of the current research study). This questionnaire is derived from previously used subjective performance measures (Vealey, 1986). It assesses **performance from the previous athletic season**.	Student athletes reported higher levels of both facets of positive body image. Significant relationships were also found between positive body image and the sport-related variables. The present results contribute novel findings to the positive body image literature and potential implications for coaches to encourage a culture that focuses less on body appearance and more on cultivating positive body image
(Stebbings et al., 2011)	* **Psychological Need Satisfaction**: Satisfaction of competence, autonomy and relatedness: ***Basic Need Satisfaction at Work Scale (BNSAW***; Deci et al., 2001) adapted to the coaching context.<br>* **Psychological Well-Being**: **coaches' positive affect and subjective vitality**:<br>- **Positive affect**: ***10-item positive affect subscale from the Positive and Negative Affect Scale*** (Watson, Tellegen, & Clark, 1988).<br>- **Coaches' subjective vitality**: ***seven-item Subjective Vitality Scale*** (Ryan & Frederick, 1997).<br>* **Coaches' perceptions of their Autonomy Supportive Behaviours**: ***six-item version of the Health Care Climate Questionnaire (HCQ***; Williams, Grow, Freedman, Ryan, & Deci, 1996) adapted to the sport context.<br>* **Coaches' perceptions of their use of controlling behaviors**:***15-item Controlling Coach Behaviors Scale (CCBS***; Bartholomew et al., 2010), modified to reflect a coach's perspective.<br>* **Social Desirability**. A ***short form of the Marlowe-Crowne social desirability***	Structural equation modelling demonstrated that coaches' competence and autonomy need satisfaction positively predicted their levels of psychological well-being, as indexed by positive affect and subjective vitality. In turn, coaches' psychological well-being positively predicted their perceived autonomy support toward their athletes, and negatively predicted their perceived controlling behaviours. Overall, the results highlight the importance of coaching contexts that facilitate coaches' psychological need satisfaction and well-being, thereby increasing the likelihood of adaptive coach interpersonal behaviour toward athletes.

	scale (Strahan & Gerbasi, 1972): participants' tendency to respond to questions in a socially desirable manner.	
(Stebbings et al., 2012)	* The **coaching context**:<br>- **opportunities for professional development** based on the *types of opportunities that have been reported as pertinent to sport coaches (Allen & Shaw, 2009*).<br>- **Job security:** ***two-item job security subscale of Chelladurai and Ogasawara's (2003)***<br>- **Coach Satisfaction** ***Questionnaire***, which was ***supplemented with two additional created items*** (e.g., "I am satisfied with my job security").<br>- **Work-life conflict**: adapted from the ***Work-Family Conflict Scale*** (Netemeyer, Boles, & McMurrian, 1996): **general sources of conflict**.<br>* **Psychological need satisfaction**. Satisfaction of **competence**, **autonomy**, and **relatedness:** ***Basic Need Satisfaction at Work Scale (BNSAW***; Deci et al., 2001) adapted to the coaching context.<br>* **Psychological need thwarting**: ***12-item Psychological Need Thwarting Scale (PNTS***; Bartholomew, Ntoumanis, Ryan, & Thøgersen-Ntoumani, 2011) adapted to the coaching context: **thwarting of coaches' psychological needs**: Competence, autonomy, and relatedness<br>* **Psychological well-being**: **coaches' positive affect and subjective vitality**.<br>- **Positive affect:** ***10-item positive affect subscale from the Positive And Negative Affect Scale (PANAS***; Watson et al., 1988).<br>* **Psychological ill-being**: **coaches' negative affect and emotional and physical exhaustion**:<br>- **Negative affect**: ***10-item negative affect subscale from the PANAS***.<br>* **Coaches' perceptions of their autonomy supportive style**. ***The six-item version of the Health Care Climate Questionnaire (HCCQ***; Williams, Grow, Freedman, Ryan, & Deci, 1996) adapted to the sport context.<br>* **Coaches' perceptions of their controlling interpersonal style:** ***15-item Controlling Coach Behaviors Scale (CCBS***; Bartholomew, et al., 2010), modified to reflect a coach's perspective.<br>* **Social desirability**. ***short form of the Marlowe-Crowne social desirability***	Controlling for socially desirable responses, structural equation modelling revealed that greater job security and opportunities for professional development, and lower work-life conflict were associated with psychological need satisfaction, which, in turn, was related to an adaptive process of psychological well-being and perceived autonomy support toward athletes. In contrast, higher work-life conflict and fewer opportunities for development were associated with a distinct maladaptive process of thwarted psychological needs, psychological ill-being, and perceived controlling interpersonal behaviour. The results highlight how the coaching context may impact upon coaches' psychological health and their interpersonal behaviour toward athletes. Moreover, evidence is provided for the independence of adaptive and maladaptive processes within the self-determination theory paradigm.

	scale (Strahan & Gerbasi, 1972).	
(Stebbings et al., 2015)	* **Positive and negative affect**: ***Positive And Negative Affect Schedule (Watson et al., 1988)***.<br>* **Integration**: extent to which coaching was personally expressive and congruent with the coaches' sense of self over the previous month: ***integration subscale*** *of the* ***Work Motivation Inventory (WMI***; Blais, Lachance, Vallerand, Briere, & Riddle, 1993) adapted to the coaching context.<br>* **Coaches' levels of devaluation:** ***five-item Devaluation subscale of the Athlete Burnout Questionnaire*** (Raedeke &Smith, 2001), adapted to the coaching context.<br>* **Coaches' perceptions of their autonomy supportive style**: ***six-item version of the Health Care Climate Questionnaire (HCCQ***; Williams, Grow, Freedman, Ryan, & Deci, 1996), adapted to the sport context.<br>* ***15-item Controlling Coach Behaviors Scale (CCBS***; Bartholomew, Ntoumanis, & Thøgersen-Ntoumani, 2010), adapted to reflect a coach's perspective: **coaches' perceptions of their controlling interpersonal style** over the previous month.<br>* **Social desirability**: ***short form of the Marlowe-Crowne Social Desirability Scale*** (Strahan & Gerbasi, 1972).	Controlling for social desirability, multilevel analyses revealed that within-person increases and individual differences in positive affect and integration were positively associated with autonomy support. Conversely, within-person increases and individual differences in negative affect, but not devaluation, were associated with increased use of interpersonal control. The indicators of well-being did not predict interpersonal control and the indicators of ill-being did not predict autonomy support. In their entirety, the present findings suggest that autonomy supportive and controlling interpersonal styles have unique correlates, and affective determinants may play a particularly central role in controlling interpersonal styles. Supporting the psychological health of coaches may lead them to create an adaptive interpersonal environment for their athletes.
(Torregrosa et al., 2014)	* **Estilo controlador**. ***Controlling Coach Behavior Scale (CCBS)*** de Bartholomew, Ntoumanis, y Thøgersen-Ntoumani (2010), validada al contexto español por Castillo et al. (2010).<br>* **Percepción de apoyo a la autonomía**: ***Escala de Apoyo a la Autonomía en Contextos de Ejercicio (PASSES)*** de Hagger et al. (2007) validada al contexto español por Moreno, Parra, y González-Cutre (2008).<br>* **Mediadores psicológicos**: ***Escala de Medición de las Necesidades Psicológicas en el Ejercicio (PNSE)*** de Wilson, Rogers, Rodgers, y Wild (2006) validada al contexto español por Moreno-Murcia, Marzo, Martínez-Galindo, y Conte (2011). tres factores: **competencia**, **autonomía**, y **relación con los demás**.<br>* **Satisfacción con la vida:** ***escala de satisfacción con la vida (ESDV-5)*** de Vallerand, Blais, Briére, y Pelletier (1989), validada al contexto español por Atienza, Pons, Balaguer, y García-Merita (2000) y Atienza, Balaguer, y García-	Linear regression analysis revealed that the controlling coach behaviour negatively affected life satisfaction, whereas autonomous support through psychological mediators positively affected life satisfaction. The results confirm the predicted effects on the life satisfaction of healthy exercise practitioners.

	Merita (2003).	
(Wayment & McDonald, 2017)	* ***developed a scale*** to assess satisfaction with key features of this unique training program.<br>- **Satisfaction with Individualized, Small-Group Training**.<br>- ***9 items from the basic psychological needs scale***: **extent to which basic psychological needs for competence, autonomy, and relatedness are met as a result of membership and participation** in the fitness studio.<br>* **Autonomous Exercise Motivation**: ***self-regulation questionnaire for exercise scale***. Four subscales were created (external regulation, introjected regulation, identified, and intrinsic).<br>* **Exercise Self-Efficacy** (ESE): ***3 items from the New General Self-Efficacy Scale***.<br>* **Well-Being**: ***5-item satisfaction with life scale***<br>* **Self-Reported Health and Energy**: ***Three items*** were averaged to form this measure.<br>* **Supportive and Self-Image Workout Goals**. ***Adapted from a measure of interpersonal goals for the exercise setting***.	In support of the basic tenets of self-determination theory, satisfaction with small-group, individualized training supported basic psychological needs, which in turn were associated with greater autonomous exercise motivation and life satisfaction. Satisfaction with this unique training method was also associated with greater exercise self-efficacy. Autonomous exercise motivation was associated with both exercise self-efficacy and greater self-reported health and energy. Discussion focuses on why exercise programs that foster a sense of social belonging (in addition to motivation and efficacy) may be helpful for successful adherence to an exercise programme.

Table S3: Study characteristics: Coach-athlete relationship and social support

REFERENCE	OBJECTIVE (extracted from the article)	STUDY DESIGN	SAMPLE: SIZE (N) + GENDER (%F/M) + AGE (R/M/SD) + SPORTS + LEVEL *R= RANGE; M= MEAN; SD= STANDARD DEVIATION*	COUNTRY OF STUDY	INTERVENTION / SETTING / TIME FRAME
(Braun et al., 2019)	To explore the strategies coaches used to try and regulate their athletes' emotions, and to explore the relationship and contextual factors influencing coaches' IER strategy use.	Qualitative Longitudinal multiple case study	N = 15: five cases, each consisting of one coach and two athletes; Age: Not reported (Varsity athletes)<br>Sport: Individual varsity sports including fencing, swimming, track and field, Nordic skiing, and squash<br>Coaches: n = 5; 100% male<br>Athletes: n = 10; 60% female; 40% male	Canada	** N/A<br>* Three weeks
(Davis & Jowett, 2014)	To examine whether athletes' attachment styles with the coach were linked to aspects of the coach-athlete relationship quality and, in turn, whether relationship quality was linked to athletes' well-being.	Quantitative Cross-sectional	N = 192; 65.5% males; 35.5% females<br>Rage = 16-32; Mage = 20.14; SD = 2.66<br>Level: university (14.6%), club (31.8%), regional (22.9%), national (17.2%), and international (12.9%)<br>Sport: Individual and team sports (e.g., netball, football, volleyball, basketball, tennis, ice skating, gymnastics, and swimming)	United Kingdom	N/A
(Dorgo et al., 2009)	To compare changes in perceived physical, mental, and social function measured by the Short Form-36 (SF36vr2) in a group of older adults who were trained by peer mentors (PMs) versus a similar group trained by qualified kinesiology student mentors (SMs).	Quantitative Longitudinal	N = 60 older adults; 48,3% female; 51,7% male<br>Rage = + 60; Mage = 68.7; SD = 6.1<br>Individuals were randomly assigned to one of the two groups:<br>- SM group (50% female; 50% male)<br>- PM group (46,7% female; 53,3% male)	United States of America	* Fitness program: SM group (trained by qualified kinesiology student mentors) & PM group (trained by peer mentors)<br>* Fitness Research Facility at the University of Texas, El Paso<br>* 14-week (Feb. 2006 to Dez. 2007)
(Felton &	To examine whether basic needs satisfaction is a	Quantitative	N = 430 athletes; 61% female; 39% male	United	N/A

Jowett, 2013a)	mechanism by which athletes' insecure attachment styles are associated with levels of well-being.	Cross-sectional	Rage = 15-35; Mage = 20.4; SD = 2.71<br>Sport: individual (59%) and team (41%) sports<br>Level: club (33%), university (20%), regional, national, and international (47%)	Kingdom	
(Felton & Jowett, 2013b)	To examine the links of the social environment, as defined by coach interpersonal behaviours and coach-athlete relationships, with athletes' psychological need satisfaction and indexes of well-being.	Quantitative Cross-sectional	N = 300 athletes; 64% female; 36% male<br>Rage = 15-30; Mage = 20.4; SD = 2.44<br>Sport: individual (41%) and team (59%) sports.<br>Level: club (32%); university (20%); regional (21%); national (17%); international (10%).	United Kingdom	N/A
(Felton & Jowett, 2015)	To examine the mediating role of basic psychological need thwarting between perceptions of athlete attachment to the coach and indexes of athlete well/ill-being.	Quantitative Cross-sectional	N = 241; 64% female; 36% male<br>Rage = 18-31 ; Mage = 20.74; SD = 2.23<br>Sports: individual (27%) and team (65%) sports<br>Level: club (7%), university (50%), regional/county (20%), and national/ international (23%)	United Kingdom	N/A
(Felton et al., 2020)	To examine the complementarity dimension of the coach-athlete relationship in relation to individual and group outcomes, specifically well-being and cohesion.	Quantitative Cross-sectional	N athletes = 304<br>**Study 1**: Athletes: n = 106; 63,2% female; 36,8% male<br>Mage = 19.91; SD = 1.54<br>Sport: more than 20 types, with the majority performed in hockey (n=18), football (n=14), rugby (n=12), and water polo (n=12).<br>Level: university (31%); club (26%); regional (23%); national (9%); international (10%); other levels of performance (1%).<br>**Study 2**: Athletes n = 198; 47% female; 53% male<br>Mage = 20.84; SD = 2.96<br>Sport: the majority participated in football (28%), rugby (20%), and netball (19%)<br>Level: university (62%); club (13%); regional (19%); national (2%); international (4%)	United Kingdom	N/A

(Katagami & Tsuchiya, 2017)	To investigate the received support experienced by university student athletes respectively from coaches and teammates over the course of a week, and examine its relationship with self-confidence and feelings of adaptation.	Quantitative Cross-sectional	N = 231 university student athletes<br>34,2% female; 65% male; 0,8% missing value<br>Mage = 19.98; SD = 0.49<br>Sports: individual sports (n=53; e.g. swimming, track and field, gymnastics, Judo, etc.), or team sports (n= 155; e.g. football, basketball, lacrosse, baseball, etc.). Missing values were 23.	Japan	N/A
(Koh et al., 2019)	To examine university coaches' implementation strategies in providing various forms of social support to their athletes.	Qualitative Interpretivist	N = 8 sport coaches; 50% female; 50% male<br>Rage = 28-70; Mage = 48.38; SD = 14.96<br>Sport: team (i.e. floorball, football, netball and handball; n = 4) and individual sports (i.e. squash, table tennis, track and field and bowling; n = 4).	Singapore	N/A
(Lafrenière et al., 2011)	To examine the role of coaches' passion for coaching in athletes' perceptions of the quality of the coach–athlete relationship.	Quantitative Cross-sectional	N = 103 coach-athlete dyads:<br>Coaches = 103; 9,7% female; 90,3% male<br>Mage = 44.23; SD = 7.94<br>Athletes n = 103; 38,8% female; 61,2% male<br>Mage = 22.04; SD = 5.29<br>Sport: e.g. gymnastics, volleyball, football, etc.<br>Level: club (N = 39; 37.9%), county (N = 5; 4.9%), university (N = 5; 4.9%), national (N = 41; 39.8%), and international (N = 13; 12.6%).	United Kingdom or French Canada	N/A
(Lafrenière et al., 2008)	To understand the role of harmonious (HP) and obsessive (OP) passion in the quality of coach-athlete relationships.	Quantitative Cross-sectional	**Study 1**: N = 157 British college athletes; 48,4% female; 51,6% male; Mage = 20.23; SD = 1.74<br>Sport: Team sports (e.g., hockey, rugby, netball).<br>Level: club (N = 10; 6%), county (N = 8; 5%), university (N = 106; 68%), national (N = 13; 8%), international (N = 20; 13%).<br>**Study 2**: N = 106 French-Canadian coaches; 8,5% female; 89,6% male; 1,9% unspecified<br>Mage = 35.48; SD = 10.83<br>Sport: e.g. gymnastics, basketball, football	United Kingdom and French Canada	N/A

(Lu et al., 2016)	To examine the conjunctive effects of athletes' resilience and coaches' social support on the relationship between life stress and burnout.	Quantitative Cross-sectional	N = 218; 27% female; 73% male<br>Rage = 18-25; Mage = 20.0; SD = 1.3<br><u>Sport</u>: individual (track and field, taekwondo, tennis, and archery; n = 162) or team sports (basketball and baseball; n = 56).<br><u>Level</u>: Division-I college student-athletes	China (Taiwan)	N/A
(Moen et al., 2019)	To study the potential mediating role of athletes' resiliency in the effect of the working alliance of the coach and the athlete on the athletes' levels of burnout.	Quantitative Cross-sectional	N = 670 athletes; 51.7% female; 49.3% male<br>Rage = 17-20; Mage = 18<br><u>Sport</u>: football (18%), handball (18%), cross country skiing (11%), biathlon (9%), ice-hockey (5%), alpine skiing (5%), cycling (5%) and track and field (4%).<br><u>Level</u>: 78% of the junior athletes in the current study had ambitions to become future elite athletes in their sports, whereas 22% did not.	Norway	N/A
(Nicholls, Levy, Jones, et al., 2016)	To assess an a priori model that included perceptions of coach behaviour, coach-athlete relationship, stress appraisals, and coping.	Quantitative Cross-sectional	N = 274 athletes; 26,6% female; 73% male; 0,4% unspecified; Rage = 16-45; Mage = 21.59; SD = 4.45<br><u>Sport</u>: team (n = 250) and individual sports (n = 24), including both contact sports (n = 216) and non-contact sports (n = 58).<br><u>Level</u>: international (n = 81), national (n = 54), county (n = 38), club (n = 36), and beginner (n = 60). Unspecified (n = 5)	United Kingdom (participants resided in the UK, Australia, and Hong Kong)	N/A
(Nicholls & Perry, 2016)	To assess dyadic coping, perceptions of relationship quality and primary stress appraisals of challenge and threat among coach-athlete dyads.	Quantitative Cross-sectional	Athletes: n = 158; 62% male; 38% female<br>Mage = 22.23; SD = 5.73<br>Coaches n = 119; 23,4% female; 76,6% male<br>Mage = 32.43; SD = 10.90<br><u>Sport</u>: team sports (132 dyads) and individual sports (26 dyads).<br><u>Level</u>: amateur (n = 123), semi-professional (n = 31), professional (n = 4)	United Kingdom	N/A
(Sagar & Jowett, 2015)	To investigate individual differences and social-contextual characteristics as predictors of fear of	Quantitative Cross-sectional	Athletes n = 367; 45% female; 55% male<br>Rage = 18-27; Mage = 20.11; SD = 1.45.	United Kingdom	N/A

	failure in the sport domain. Specifically, it examined: (1) self-control and socio-contextual characteristics in the quality of relationships between coaches and athletes as potential correlates of fear of failure; and (2), the capacity of self-control and relationship quality to predict the undesired behavioural tendencies of athletes' fear of failure.		Sport: a variety of team and individual sports: rugby, athletics (10% each), American football, basketball, gymnastics, soccer (9% each), swimming, netball, rowing, volleyball, triathlon, tennis, badminton (7% each), and judo (2%)<br>Level: regional/county (65%), national (19%), international (16%)		
(Staff et al., 2017)	To qualitatively explore coping from an interpersonal perspective (i.e., dyadic coping) in coach-athlete relationships.	Qualitative Multiple case study	5 coach-athlete dyads:<br>Coaches n = 5; Mage = 37.65; SD = 10.07<br>Athletes n = 5; Mage = 21.85; SD = 2.92<br>Sport: individual sports (track and field, n = 3; squash, n = 1; triathlon, n = 1; swiMixed Methodsing, n = 1).<br>Level: University competition or above	United Kingdom	N/A
(Stefansen et al., 2019)	To explore young athletes' thinking about coach-athlete sexual relationships (CASRs); to further the understanding of the ambivalence surrounding CASRs in the sports field; to analyse how athletes understand and justify CASRs.	Qualitative	N = 106 sport students; gender mixed (no numbers reported); Rage = 19–26	Norway	N/A
(Trouilloud & Amiel, 2011)	To evaluate how the reflected appraisals, the perception of how athletes are viewed by coaches, parents and teammates, affects athletes' self-perception.	Quantitative Cross-sectional	N = 372 athletes; 41,4% female; 58,6% male<br>Rage = 18-40; Mage = 21.03; SD = 2.81.<br>Sport: team sports (n = 165; 44.35%), tennis (n = 50; 13.44%), track and field (n = 29; 7.80%), swimming (n = 22; 5.91%), gymnastics (n = 21; 5.65 %), fighting sports (n = 20; 5.38%), skiing (n = 19; 5.11%), and diverse others sports (n = 46; 12.36%).<br>Level: local (n = 120; 32.26%), regional (n = 143; 38.44%) national (n = 109; 29.30%).	France	N/A
(van Kleef et al., 2019)	To examine how coaches' emotional expressions influence players' affect, cognition, and behaviour.	Quantitative<br>* Study 1: cross-sectional<br>* Study 2: cross-lagged with three	**Study 1**: Sport: baseball/softball<br>Coaches: n = 29 (one coach returned an empty questionnaire); 14% female; 86% male<br>Mage = 48.03; SD = 10.26<br>Players n = 268; 34.6% female; 61.2% male; one athlete	The Netherlands	** N/A<br>* three measurement points (before, during, and after the game)

		measurement points (before, during, and after the game).	did not disclose gender information.<br>Mage = 27.64; SD = 10.29<br>**Study 2**: Sport: soccer<br>Coaches n = 30; 100% male; Mage of 41.17; SD = 11.67<br>Players n = 376 players; 10.4% female; 89.6% male<br>Mage = 21.05; SD = 5.50		

Table S4: Key findings: Coach-athlete relationship and social support

REFERENCE	CATEGORIES ANALISED AND MEASUREMENT INSTRUMENTS	KEY FINDINGS REPORTED (extracted from the article)
(Braun et al., 2019)	* What **Interpersonal Emotion Regulation (IER) strategies do coaches use to try and regulate the emotions of their athletes**?<br>* **How does the coach-athlete relationship influence IER among coaches and athletes**?<br>* **What contextual features influence IER among coaches and athletes**?<br><br>***individual interviews*** + ***audio diary*** period	Participants described a bidirectional association between the coach-athlete relationship and coaches' IER. A number of factors influenced athletes' and coaches' use of emotion regulation strategies and contributed to the quality of the coach-athlete relationship. The IER strategies that coaches used may reflect instrumental, performance-related motives, and coaches' IER efforts may also contribute to coaches' emotional labour
(Davis & Jowett, 2014)	***Questionnaire*** measuring:<br>*** Athletes' attachment styles with the coach**<br>*** Athletes' relationship quality with the coach**<br>*** Athletes' feelings of positive affect (PA) and negative affect (NA).**	Structural equation modelling (SEM) analysis found athletes' avoidant and secure attachment styles to be associated with aspects of coach-athlete relationship quality such as social support, relationship depth, and interpersonal conflict. Interpersonal conflict appeared to play a key role in athletes' PA and NA. From a practical perspective, an understanding of conflict management could provide a resource that allows athletes (and coaches) to enhance the quality of their sporting relationships. Specifically, an awareness of proactive strategies (e.g., steps to clarify expectations) and reactive strategies (e.g., cooperation during the discussion of disagreements) could potentially lead both coaches and athletes to "broaden" their viewpoints and in turn "build" connections that are capable of generating positive emotions including interest, excitement, happiness, and zeal.
(Dorgo et al., 2009)	**Overall functioning** defined as **perceived physical, mental, and social function**: ***SF-36vr2***: two summary scores and scores for eight individual scales:<br>- **physical component summary or PCS**. PCS is a composite of:<br>***physical functioning (PF)**<br>***role physical (RP)**<br>***bodily pain (BP)**<br>***general health (GH)**<br>- **mental component summary or MCS**. MCS includes:	After a 14-week physical fitness intervention, perceived physical, mental, and social functioning improved significantly ($p < .05$) for the PM group, but not for the SM group ($p > .06$). Thus, older adults who participated in a physical fitness program with peer support perceived (a) overall improvement in physical and mental well-being; (b) better social functioning, (c) enhanced ability to carry out physical and emotional roles, (d) improved general health, and (e) increased level of vitality. Peer-mentored exercise programs for

	vitality (VT)**<br>social functioning (SF)**<br>***role emotional (RE)**<br>***mental health (MH)**	older adults are superior to programs mentored by young professionals and may lead to increased adherence.
(Felton & Jowett, 2013a)	* ***Experiences in Close Relationships Scale e Short version (ECR-S**; Wei, Russell, Mallinckrodt, & Vogel, 2007): **athlete's attachment style** by assessing **how they generally experience close relationships**.<br>* ***Need Satisfaction Scale (NSS**; La Guardia et al., 2000): the **degree to which the basic psychological needs of the athlete were satisfied within the coach-athlete relational context and the parent-athlete relational context**.<br>* ***Subjective Vitality Scale (SVS**; Ryan & Frederick., 1997). The SVS is a 7-item measure that assesses **perceptions of mental and physical aliveness and energy** in general terms.<br>* ***Rosenberg Self-Esteem Scale (RSE**; Rosenberg., 1965). The RSE scale is a 10-item measure that assesses **individual's perception relative to how they regard themselves**.<br>* ***Elite Athlete Self Description Questionnaire (EASDQ**; Marsh, Hey, Johnson, & Perry, 1997): athletes' perceptions of their physical self-concept across five dimensions; skill ability, body shape, physiological state, mental competence, and overall performance. For the purpose of this study only the sub-scales of skill ability and overall performance self-concept were employed.<br>* ***The International Positive and Negative Affect Schedule - Short Form (I-PANAS-SF**; Thompson, 2007). This scale was employed to assess the level of positive and negative affect experienced by the athletes.	Bootstrap mediation analysis revealed that athletes' perceptions of satisfaction of basic psychological needs generally mediated the association between their attachment styles and well-being. Moreover, the indirect effect of athletes' experience of the satisfaction of basic needs on well-being was greater within the parental relational context than within the coaching relational context. Overall, the findings from the study highlight that the integration of attachment and self-determination theories can promote understanding of relational process in sport.
(Felton & Jowett, 2013b)	* ***Coach–Athlete Relationship Questionnaire (CART-Q**; Jowett & Ntoumanis, 2004): **perceptions of the coach–athlete relationship quality**; athletes' direct perceptions of: **closeness, commitment** and **complementarity.**<br>* ***Sport Climate Questionnaire (SCQ**; Reinboth et al., 2004): **degree to which a coach is autonomy supportive**.<br>* ***Coaches' Controlling Behavior Scale (CCBS**; Bartholomew et al., 2010): **athletes' perceptions of their coaches controlling behavior**<br>* ***Need Satisfaction Scale (NSS**; La Guardia et al., 2000): **satisfaction of the**	Bootstrap mediation analysis highlighted significant indirect effects whereby the competence need mediated associations between the social environment of coaching and athletes' vitality, negative affect, and physical self-concept (defined as skilfulness and performance). Findings support theoretical assumptions and highlight that athletes' perceptions of what coaches do, and how they relate, are important to their psychological needs satisfaction and optimal functioning.

	**three basic needs** (**autonomy, competence, and relatedness**).<br>* ***Subjective Vitality Scale (SVS***; Ryan & Frederick, 1997): **perceptions of mental and physical aliveness and energy** in general terms.<br>* ***Elite Athlete Self-Description Questionnaire (EASDQ***; Marsh et al., 1997): **athletes' perceptions of physical self-concept** across five dimensions: **skill ability, body shape, physiological state, mental competence,** and **overall performance**.<br>* *Only* ***five negative affect items from*** The ***International Positive and Negative Affect Schedule –Short Form (I-PANAS-SF***; Thompson, 2007): **level of positive and negative affect experienced** by the individuals: **athletes' level of ill-being**.	
(Felton & Jowett, 2015)	* ***Psychological Need Thwarting Scale (PNTS)*** (Bartholomew et al., 2011b)**, within the coaching relational and sport contexts**.<br>* ***Coach–Athlete Attachment Scale (CAAS)***: it measures an **athlete's avoidant**, **anxious**, and **secure attachment**.<br>* ***Satisfaction with Life Scale (SLS)***<br>* ***Athlete Satisfaction Questionnaire (ASQ)*** (Riemer & Chelladurai, 1998): The **degree of performance satisfaction perceived by the athletes**.<br>* ***Depression subscale of the Brief Symptom Inventory (BSI)*** (Derogatis & Melisaratos, 1983): **Depression**<br>* ***negative affect subscale of the International Positive and Negative Affect Scale-Short Form (I-PANAS-SF)*** (Thompson, 2007). The **athletes' experience of negative affect**.	Bootstrap mediation analysis revealed that the perceived psychological needs of thwarted autonomy and competence within the coach relational context mediated the associations between athletes' perceptions of insecure attachments to the coach and experiences of life satisfaction and negative affect. Analysis also revealed that the perceived psychological needs of thwarted competence and relatedness within the sport context mediated the associations between athletes' attachment style and experiences of performance satisfaction, life satisfaction, depression, and negative affect. Overall, the findings of the study highlight that the examination of negative aspects of sport participation may facilitate a more complete understanding of athletes' psychological functioning.
(Felton et al., 2020)	* The ***Complementarity dimension of the Coach-Athlete Relationship Questionnaire (CART-Q***; Jowett, 2009; Jowett & Ntoumanis, 2004): **athletes' interpersonal behaviours**. Both **athletes' direct perspective of corresponding complementarity,** and **athletes' meta-perspective of corresponding complementarity** were assessed.<br>* The ***Basic Need Satisfaction in Relationships Questionnaire (BNSRQ***; La Guardia, Ryan, Couchman, & Deci, 2000): the **extent athletes' basic psychological needs were satisfied within the context of the coach-athlete**	In Study 1 (n = 106), mediation analysis demonstrated significant indirect effects between direct- and meta complementarity and vitality via basic psychological needs satisfaction. In addition, a significant direct effect between direct complementarity and vitality was also seen, independent of the indirect effect.<br>In Study 2 (n = 198), mediation analysis demonstrated significant indirect effects between direct- and meta complementarity and task and social cohesion via the basic psychological needs. A significant

	**relationship**.<br>* *Subjective Vitality Scale (SVS*; Ryan and Frederick, 1997): **athletes' mental and physical vitality**	direct effect between meta complementarity and task cohesion was also identified, independent of the indirect effects. No direct or indirect effects were observed for reciprocal complementarity. Findings highlight the importance of complementarity, and satisfaction of the basic psychological needs, within the coach-athlete relationship for enhancing athletes' feelings of well-being and cohesion.
(Katagami & Tsuchiya, 2017)	* *Japanese version of the Athlete Received Support Questionnaire (ARSQ-J*: Katagami and Tsuchiya, 2015): **received support experienced by university student athletes** respectively from coaches and teammates and **its relationship with self-confidence and feelings of adaptation**.<br>* *Competitive State Anxiety Inventory-2 (the CSAI-2R*: Cox et al., 2003): **self-confidence in a sports context**<br>* **Feelings of adaptation**: a ***self-esteem scale (Yamamoto et al., 1982)***. Self-esteem is considered as an indicator of an individual's feelings of adaptation to the current situation	The results indicated that received support might influence recipients' self-confidence both positively and negatively, depending on its dimensions and providers. Esteem support both from coaches and teammates were effective for self-confidence. Tangible support had a positive impact if provided from teammates, but a negative impact if provided by coaches. With regard to the feelings of adaptation, it was indicated that tangible support both from coaches and teammates were negatively correlated with the outcome. In conclusion, its dimension and provider of support can determine the effectiveness of social support.
(Koh et al., 2019)	* University coaches' **implementation strategies in providing social support** to their athletes: ***Interviews***.	The results revealed that coaches from different sports shared similar strategies across emotional, esteem, informational and tangible dimensions, but with some distinguishable differences in the way these strategies were implemented. In documenting the lived experiences of sport coaches, key strategies valued highly among these coaches were highlighted, providing important implications for coaches to know how to incorporate these strategies into their coaching practice to better support athletes' well-being and improve the quality of coaching. The findings also provide an implementation framework of social support that emphasizes key strategies for coaches to focus on in their coaching approaches.

(Lafrenière et al., 2011)	* **Coaches' harmonious and obsessive passion for coaching**: ***adapted version of the Passion Scale*** (Vallerand et al., 2003),<br>* **Coaches' autonomy-supportive behaviors toward their athletes**. *¿...?*<br>* **Coaches' controlling behaviors toward their athlete**. *¿...?*<br>* **Athletes' perceptions of the quality of the relationship with their coach (closeness, commitment, and complementarity** corresponding to the **affective, cognitive, and behavioural aspects of the coach-athlete relationship**, respectively): ***CART-Q***<br>* **Athletes' happiness (in terms of positive affect)**. The ***positive affect subscale*** of the ***short Positive and Negative Affect Schedule (PANAS***; Mackinnon et al., 1999; Watson, Clark, & Tellegen, 1988)	Results from structural equation modelling revealed that harmonious passion for coaching positively predicted autonomy-supportive behaviours toward their athletes, while obsessive passion for coaching positively predicted controlling behaviours. Moreover, autonomy-supportive behaviours predicted high quality coach–athlete relationships as perceived by athletes that, in turn, positively predicted athletes' general happiness. This study provides insights into the psychological factors that allow coaches to instigate high quality relationships with their athletes and the impact of the relationship on athletes' general happiness.
(Lafrenière et al., 2008)	* **Athletes' harmonious and obsessive passion toward sport: *Passion Scale (Vallerand et al., 2003) (Study 1 & 2)***<br>* The ***Coach-Athlete Relationship Questionnaire (CART-Q***; Jowett & Ntoumanis, 2004) ***(Study 1)***:<br>- the **athletes' direct perspective of the quality of the relationship with their coach**.<br>- the meta-perspective of the quality of the relationship with their coach: **how athletes believe their coach perceives their relationship**.<br>* The ***Interpersonal Relationship Quality Scale*** (Senécal, Vallerand, & Vallières, 1992) ***(Study 2)***: **quality of the coach–athlete relationship**.<br>* **Coaches' situational positive emotions *(Study 2): three items*** assessing positive emotions taken from ***Barrett and Russell (1998)***.<br>* **Satisfaction With Life *(Study 2): four items*** from the ***Satisfaction with Life Scale*** (Blais, Vallerand, Pelletier, & Brière, 1989).<br>* Coaches' **General Positive and Negative Affect** generally experienced in life ***(Study 2)***: ***short PANAS scales*** (Watson, Clark, & Tellegen, 1988).	Results of **Study 1**, conducted with **athletes** (N = 157), revealed that HP positively predicts a high-quality coach-athlete relationship, whereas OP was largely unrelated to such relationships. **Study 2** was conducted with **coaches** (N = 106) and showed that only HP positively predicted the quality of the coach-athlete relationship. Furthermore, these effects were fully mediated by positive emotions. Finally, the quality of the coach-athlete relationship positively predicted coaches' subjective well-being.
(Lu et al., 2016)	* **Life stress**: ***24-item College Student-Athlete Life Stress Scale (CSALSS***; Lu, Hsu, Chan, Cheen, & Kao, 2012). eight subscales for **general life stressors** (academic requirements, family relationships, interpersonal relationships, romantic relationships) and **sport life stressors** (coach relationships, performance demand, sports injury, training adaptation).	Resilience and coaches' social support conjunctively moderated the stress-burnout relationship. Specifically the interaction of athletes' resilience with coaches' informational and tangible social support moderated athletes' stress-burnout relationship in high and low life stress conditions.

	* Individuals' **resilience** as a personality trait: ***abbreviated version of the Connor-Davidson Resilience Scale e 2 (CD-RISC2***; Vaishnavi, Connor, & Davidson, 2007), taken from the original CDeRISC (Connor & Davidson, 2003).<br>* ***Athletes' Received Support Questionnaire (ARSQ***; Freeman, Coffee, Moll, Rees, &Sammy, 2014): four types of **social support that athletes received (**slightly modified to measure coaches' social support**)**: **emotional support**, **esteem support**, **informational support**, and **tangible support**.<br>* Athletes' **burnout** symptoms**:** ***Athlete Burnout Questionnaire (ABQ***; Raedeke & Smith, 2001): three subscales: emotional or physical exhaustion, reduced sense of accomplishment, and sport devaluation.	
(Moen et al., 2019)	Norwegian version of all scales:<br>* ***The Working Alliance Inventory (WAI***; Horvath & Greenberg, 1989; Tracey & Kokotovic, 1989) adjusted for the sport context: **coach– athlete relationship characteristics**. Three central dimensions: (a) **agreement on the goals**; (b) **agreement on**; and (c) the **development of a personal bond** between the coach and the athlete.<br>* ***The Resilience scale for adults (RSA)***: six **protective dimensions of resilience in adults**: (1) Perception of the Self, (2) Planned Future, (3) Social Competence, (4) Family Cohesion, (5) Social Resources, (6) Structured Style (Friborg & Hjemdal, 2004; Friborg, Hjemdal, Rosenvinge, & Martinussen, 2003; Hjemdal, Friborg, Martinussen, & Rosenvinge, 2001).<br>* ***The Athlete Burnout Questionnaire*** (***ABQ***; Raedeke & Smith, 2001, 2009): **athlete burnout**: three key dimensions of burnout: (1) devaluation of sports participation, (2) a reduced sense of accomplishment, and (3) emotional and physical exhaustion.	Structural equation modelling analyses showed a strong positive association between the coach-athlete working alliance and resilience, and a strong negative association between resilience and burnout. A moderate association was found between the coach-athlete working alliance athlete burnout. Furthermore, a mediational relationship was detected, where the effect of working alliance on less burnout partly was explained by an indirect effect of working alliance through more resilience in the athlete. The current study highlights the importance of a strong working alliance between coaches and athletes to develop athlete resilience, and the importance of athlete resilience as a buffer to prevent occurences of athlete burnout.
(Nicholls & Perry, 2016)	* ***Coach and athlete version of the Dyadic Coping Inventory (DCI***; Levesque et al., 2014): **positive and negative dyadic coping**.<br>* The ***Coach Athlete Relationship Questionnaire (CART-Q***; Jowett and Ntoumanis, 2004): **athletes' and coaches overall perceptions of relationship quality**: **closeness, commitment, and complementarity**.<br>* **Primary Stress Appraisals**: ***four challenge*** and ***four threat questions*** from the ***Stress Appraisal Measure (SAM***; Peacock and Wong, 1990).	These actor-partner analyses revealed differences between athletes and coaches. Although the actor effects were relatively large compared to partner effects, perceptions of relationship quality demonstrated little impact on athletes. The mediating role of relationship quality was broadly as important as dyadic coping for coaches. These findings provide an insight in to how coach-athlete dyads interact to manage stress and indicate that relationship quality

		is of particular importance for coaches, but less important for athletes. In order to improve perceptions of relationship quality among coaches and athletes, interventions could be developed to foster positive dyadic coping among both coaches and athletes, which may also impact upon stress appraisals of challenge and threat.
(Nicholls, Levy, Jones, et al., 2016)	* **Athletes' perceptions of coach behaviour**: ***47-item CBS11***<br>* **Coach–athlete relationship**: ***11-item Coach Athlete Relationship*** *Questionnaire (CART-Q)*: **athletes' perceptions of closeness**, **commitment** and **complementarity with their coach**.<br>* *Stress Appraisal Measure (SAM):* **three primary appraisals (i.e. challenge, threat, and centrality), three secondary appraisals (controllable-by-self, controllable-by-others, and uncontrollable-by-anyone), and stressfulness (i.e. overall feeling of stress).**<br>* *Coping Inventory for Competitive Sport (CICS):* **how the athletes were coping before their competition**.	The results revealed that perceptions of coach behaviour were associated with aspects of the coach-athlete relationship and stress appraisals. In particular, closeness was positively associated with challenge appraisals and negatively with threat appraisals. However, commitment was positively associated with threat, indicating that there might be some negative implications of having a highly committed coach--athlete relationship. Further, commitment was also positively associated with disengagement-oriented coping, which has previously been linked to poor performance and lower goal-attainment.
(Sagar & Jowett, 2015)	* **Fear of failure (beliefs associated with aversive consequences of failure)**: *Performance Failure Appraisal Inventory - PFAI* (Conroy et al., 2002): five subscales capturing **fear of experiencing shame and embarrassment**, **fear of having an uncertain future**, **fear of devaluing one's self-estimate**, **fear of important others losing interest**, and **fear of upsetting important others**<br>* **Trait self-control**: ***13-item Brief Self-Control Scale (Tangney et al., 2004)***: **spheres of self-control that relate to control over thoughts and emotions**, **impulse control**, **performance regulation**, **and habit breaking**.<br>* **Coach-athlete relationship quality**: *Coach-Athlete Relationship Questionnaire (CART-Q*; Jowett & Ntoumanis, 2004): **athletes' perceptions of closeness**, **commitment**, **and complementarity with their coach.**<br>* **Athletes' perception of coach empathy**: ***eight positively worded items from the empathy subscale of the Relationship Inventory***	Multiple regression analyses revealed that self-control and empathy predicted both interpersonal and intrapersonal dimensions of fear of failure, whereas relationship quality predicted only interpersonal dimensions of fear of failure. Self-control acted as a beneficial regulatory strategy to diffuse intra- and inter-personal types of fear of failure. Self-control, empathy and relationship quality appear to be likely predictors or antecedents of fear of failure. Thus, it would seem that the development of quality relationships characterised by affective closeness, commitment, complementary transactions and empathy, as well as the possession of self-control are key factors in reducing fear of failure among individuals.
(Staff et al., 2017)	* **Dyadic coping in coach-athlete relationships**:<br>- **Essence of dyadic copin**g: coaches' and athletes' understanding of dyadic coping	Five themes were identified. These represented the essence of dyadic coping, antecedents of dyadic coping (themes: lock and key fit, friendship and trust, communication of the stressor), and outcomes

	- **Antecedents of dyadic coping**: the factors that were necessary for dyadic coping to occur: lock and key fit, friendship and trust, communication of the stressor<br>- **Outcomes of dyadic coping**: protection and support: the positive nurturing environment<br>***individual interviews***	of dyadic coping (theme: protection and support). The first theme captures coaches' and athletes' understanding of dyadic coping. The antecedent themes represent the factors that were necessary for dyadic coping to occur. Protection and support relates to the positive nurturing environment that was discussed as an outcome of dyadic coping. The findings highlight that dyadic coping was prevalent in coach-athlete relationships when various antecedents (lock and key fit, friendship and trust, communication of the stressor) existed. Protection and support were pertinent outcomes of dyadic coping that contributed to personal and relationship growth.
(Stefansen et al., 2019)	*** The Safeguarding Ethic: An Athlete's: Need for Protection**<br>*** The Love Ethic: Feelings Cannot be Regulated**<br>*** The Athletic-Performance Ethic: Preserving the Sport "Contract"**<br>**- athletes' understandings of CASR: 20 gender-mixed focus group interviews**<br>**- one-page questionnaire: background information on the participants, including their sport and coaching experiences.**<br>**- "video elicitation interview" approach (e.g., Henry & Fetters, 2012) using four short films to launch discussions.**	Three different ethics were activated in the interviews: the safeguarding, love, and athletic-performance ethics. These ethics are linked to different underlying "imaginaries," or cultural frames, about the meaning of sport in society. The results can inform sporting organizations' future prevention efforts.
(Trouilloud & Amiel, 2011)	* **Reflected appraisals of coaches, parents and teammates**. Athletes' perceptions of significant others' beliefs were assessed distinctly for coaches, parents and teammates: ***12-item scale adapted from Amorose's scale (2003)***:<br>- **Reflected appraisals about athletes' competence** and about **athletes' capacity to make progress in the future**.<br>- **Athletes' self-perceptions of** sport **competence and future progress**.<br>- **Background characteristics**: ***socio-demographic items***	A survey of young adult athletes finds that not only did a positive perception of reflected appraisals affect athletes' self-evaluation of their own competence, but also positively affected their belief in future progress in their sport.
(van Kleef et al., 2019)	**Effects of coaches' emotional expressions on players' affect, cognition, and behaviour:**<br>*** Effects of coaches' emotional expressions on players' emotional experience**<br>*** Effects of coaches' emotional expressions on players' inferences regarding the quality of their performance**<br>*** Effects of coaches' emotional expressions on team performance**	Coaches' expressions of happiness and anger predicted (1) players' experiences of happiness and anger, (2) players' inferences about the quality of their performance, and (3) objective team performance outcomes. Regarding team performance, results indicated that coaches' expressions of happiness were conducive to team performance, whereas expressions of anger were not.
